# An Adhesion-Dependent Switch between Mechanisms That Determine Motile Cell Shape

**DOI:** 10.1371/journal.pbio.1001059

**Published:** 2011-05-03

**Authors:** Erin L. Barnhart, Kun-Chun Lee, Kinneret Keren, Alex Mogilner, Julie A. Theriot

**Affiliations:** 1 Department of Biochemistry and Howard Hughes Medical Institute, Stanford School of Medicine, Stanford, California, United States of America; 2 Department of Mathematics, University of California, Davis, California, United States of America; 3 Department of Physics and Russell Berrie Nanotechnology Institute, Technion – Israel Institute of Technology, Haifa, Israel; 4 Department of Microbiology and Immunology, Stanford School of Medicine, Stanford, California, United States of America; University of Washington, United States of America

## Abstract

Keratocytes are fast-moving cells in which adhesion dynamics are tightly coupled to the actin polymerization motor that drives migration, resulting in highly coordinated cell movement. We have found that modifying the adhesive properties of the underlying substrate has a dramatic effect on keratocyte morphology. Cells crawling at intermediate adhesion strengths resembled stereotypical keratocytes, characterized by a broad, fan-shaped lamellipodium, clearly defined leading and trailing edges, and persistent rates of protrusion and retraction. Cells at low adhesion strength were small and round with highly variable protrusion and retraction rates, and cells at high adhesion strength were large and asymmetrical and, strikingly, exhibited traveling waves of protrusion. To elucidate the mechanisms by which adhesion strength determines cell behavior, we examined the organization of adhesions, myosin II, and the actin network in keratocytes migrating on substrates with different adhesion strengths. On the whole, our results are consistent with a quantitative physical model in which keratocyte shape and migratory behavior emerge from the self-organization of actin, adhesions, and myosin, and quantitative changes in either adhesion strength or myosin contraction can switch keratocytes among qualitatively distinct migration regimes.

## Author Summary

Cell migration is important for many biological processes: white blood cells chase down and kill bacteria to guard against infection, epithelial cells crawl across open wounds to promote healing, and embryonic cells move collectively to form organs and tissues during embryogenesis. In all of these cases, migration depends on the spatial and temporal organization of multiple forces, including actin-driven protrusion of the cell membrane, membrane tension, cell-substrate adhesion, and myosin-mediated contraction of the actin network. In this work, we have used a simple cell type, the fish epithelial keratocyte, as a model system to investigate the manner in which these forces are integrated to give rise to large-scale emergent properties such as cell shape and movement. Keratocytes are normally fan-shaped and fast-moving, but we have found that keratocytes migrate more slowly and assume round or asymmetric shapes when cell-substrate adhesion strength is too high or too low. By correlating measurements of adhesion-dependent changes in cell shape and speed with measurements of adhesion and myosin localization patterns and actin network organization, we have developed a mechanical model in which keratocyte shape and movement emerge from adhesion and myosin-dependent regulation of the dynamic actin cytoskeleton.

## Introduction

Motile cell shape and speed emerge from nanometer-scale interactions among constituent elements, including the actin network, myosin, adhesions, and the cell membrane [1]. Cell-substrate adhesion strength has a dramatic, biphasic effect on cell migration velocity: cell speed increases between low and intermediate adhesion strengths and decreases between intermediate and high adhesion strengths [2]–[5]. In addition, optimal adhesion strength for fast cell migration has been shown to depend on the level of myosin contraction, with cells crawling at faster speeds at low and high adhesion strengths when myosin activity is decreased or increased, respectively [3]. Thus, the balance between adhesion and myosin contraction clearly contributes to determining cell speed. However, the degree to which adhesion strength and myosin contraction may contribute to other properties of motile cells such as cell shape is poorly characterized.

Adhesion strength and myosin contraction have mechanical consequences that are likely to affect cell shape determination. Cells are thought to transmit forces to the underlying substrate via a mechanism in which adhesions act as “molecular clutches” that couple the actin network to the substrate [6]. This physical linkage creates a frictional slippage interface that balances myosin-mediated contractile forces [7]–[11]. According to this model, as the number of clutches increases, the friction coefficient increases, increasing the amount of traction force that can be transmitted to the surface and slowing retrograde flow of the actin network. In contrast, as the amount of myosin contraction, and the amount of force transmitted by engaged clutches, increases, the off-rate constant for the clutches increases exponentially [12], reducing the average lifetime for the population of clutches. This effectively decreases the coefficient of friction between the cell and the substrate, reducing the amount of traction force that can be transmitted to the substrate and increasing actin retrograde flow. The dynamics of the cell boundary, and therefore cell shape, are determined in part by adhesion- and myosin-dependent friction and retrograde flow rates: high friction stabilizes actin-driven protrusion of the cell boundary, whereas low friction results in retrograde flow of the actin network and retraction of the cell boundary.

In addition to these mechanical effects, adhesion strength and myosin contraction affect organization of the actin network through a variety of signal transduction pathways [3]. Adhesions are complex, hierarchical structures: integrin molecules bind extracellular matrix proteins on the underlying surface, and many additional adhesion proteins, including proteins involved in signal transduction and actin binding proteins, recruit to adhesions on the inside of the cell [13]. Thus, these complex adhesions act as organizing centers, localizing biochemical signals that modify the organization of the actin network. For example, some evidence suggests that nascent adhesions activate Rac GTPase [14], which promotes branching and polymerization of the actin network by activating Arp2/3 [15]–[17]. Mature adhesions, on the other hand, are thought to up-regulate RhoA GTPase activity [18],[19], which promotes bundling of the actin network by activating the formin mDia1 [20]. RhoA also promotes myosin contraction by up-regulating myosin light chain kinase [21], and myosin contraction, in turn, promotes adhesion maturation [22]–[24], bundling of actin filaments [25], and actin depolymerization [26]. The manner in which mechanical and biochemical feedback among adhesions, myosin, and actin contributes to global actin network organization and cell shape determination is not well understood.

Fish epithelial keratocytes are an ideal model system for investigating cell shape determination [1],[27],[28]. Individual keratocytes maintain nearly constant shape, speed, and direction over many cell lengths of migration, but there is considerable heterogeneity within a population of keratocytes [29]–[33]. New methods for quantifying cell shape [34] have facilitated correlative studies of shape and actin network organization in large populations of keratocytes [29],[30], resulting in a model for shape determination based on mechanical feedback between the treadmilling actin network and the inextensible cell membrane [29]. In this model, the polymerizing actin network pushes on the cell membrane from within, generating membrane tension that rapidly equilibrates and exerts globally constant force, per unit length, on the actin network. At the center of the leading edge, high actin filament density results in low membrane resistance per filament, allowing actin filaments to polymerize rapidly and drive protrusion of the leading edge. As the filament density decreases towards the cell sides, resistance per filament increases until the load due to membrane tension stalls actin polymerization, thereby setting the front corners of the cell. This model is consistent with experimental evidence that actin network densities are graded in fan-shaped keratocytes, but does not explicitly address the contributions of adhesions and myosin to the establishment of this graded actin filament distribution and overall cell shape.

In this work, we investigated the contributions of adhesion and myosin contraction to dynamic actin network organization and keratocyte shape determination. We found that keratocyte shape and speed both have a biphasic dependence on adhesion strength; keratocytes crawling at intermediate adhesion strength are fast and fan-shaped, whereas keratocytes crawling at low and high adhesion strengths are slow and round. To elucidate the mechanism of adhesion-dependent shape determination, we examined actin network organization and dynamics, myosin localization, and adhesion distribution, as well as the consequences of myosin inhibition or activation, for cells plated at low, intermediate, or high adhesion strengths. We present a quantitative mechanical model in which adhesion-dependent actin polymerization and retrograde flow rates add vectorially at each point around the perimeter of the cell, determining cell shape. We find that as adhesion strength increases, cells undergo a qualitative switch in the mechanism of shape determination: at low adhesion strength, the pattern of actin retrograde flow is most important, while at high adhesion strength, the pattern of actin network growth becomes dominant. Overall, our mechanical model for cell shape determination is able to integrate the effects of all the known major relevant cellular components to generate quantitative predictions for large-scale cell behavior.

## Results

### Effects of Substrate Adhesivity on Keratocyte Shape and Movement

**Figure 1:**
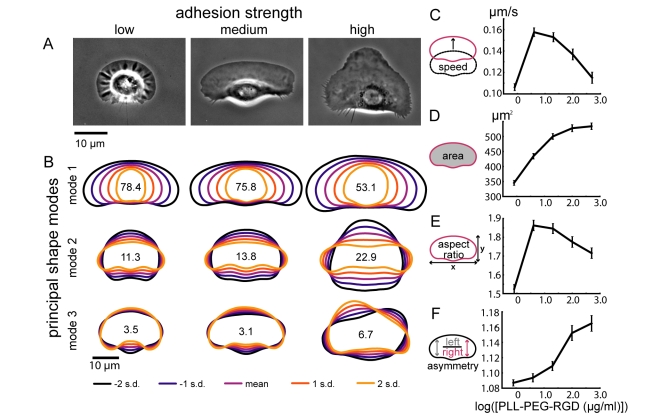
Adhesion strength of the underlying surface affects keratocyte migration speed and shape. (A) Phase contrast images of representative cells crawling at low (left), intermediate (center), and high (right) adhesion strengths (0.8, 4, and 500 µg/ml PLL-PEG-RGD, respectively). (B) Principal modes of shape variation, as determined by principal component analysis of aligned cell outlines, are shown for populations of cells at low (left), intermediate (center), and high (right) adhesion strengths (n>200 cells for each population). For each population of cells, the mean cell shape and shapes one and two standard deviations from the mean are shown for each shape mode. The variation accounted for by each mode is indicated. (C–F) Average cell speed (C), area (D), aspect ratio (E), and left-right asymmetry (F) are shown for live cells plated on surfaces coated with the indicated PLL-PEG-RGD concentrations. Error bars indicate standard error of the mean.

To determine the effect of adhesion strength on keratocyte motility, we modified the adhesive properties of the underlying substrate by using an Arg-Gly-Asp (RGD) functionalized poly-L-lysine-graft-(polyethylene glycol) copolymer (PLL-PEG-RGD) [35]. The positively charged PLL backbone of the copolymer binds negatively charged glass surfaces, the PEG chains prevent non-specific adsorption of serum proteins to the surface, and the RGD peptides promote specific cell adhesion via integrin binding [36],[37]. Keratocytes were plated on glass surfaces coated with a range of RGD densities, where the RGD density was controlled by dilution of the PLL-PEG-RGD copolymer with a non-functionalized PLL-PEG copolymer. The strength of cell-substrate attachment increased with increasing PLL-PEG-RGD concentration, as expected (Figure S1), so for simplicity we refer to cells migrating on substrates coated with varying concentrations of PLL-PEG-RGD as migrating at various “adhesion strengths.” We found that keratocyte migration speed exhibited a biphasic dependence on adhesion strength (Figure 1C) similar to that previously observed in several slow-moving cell types [2]–[5]: cells plated at intermediate adhesion strengths migrated at faster speeds than cells plated at low and high adhesion strengths (0.16 µm/s on surfaces coated with 4 µg/ml PLL-PEG-RGD, compared to 0.12 and 0.11 µm/s on surfaces coated with 0.8 and 500 µg/ml PLL-PEG-RGD, respectively). Compared to other cell types, however, the dependence of keratocyte speed on adhesion strength was modest, with less than 50% change in migration speed across a wide range of adhesion strengths (Figure 1C).

**Figure 2:**
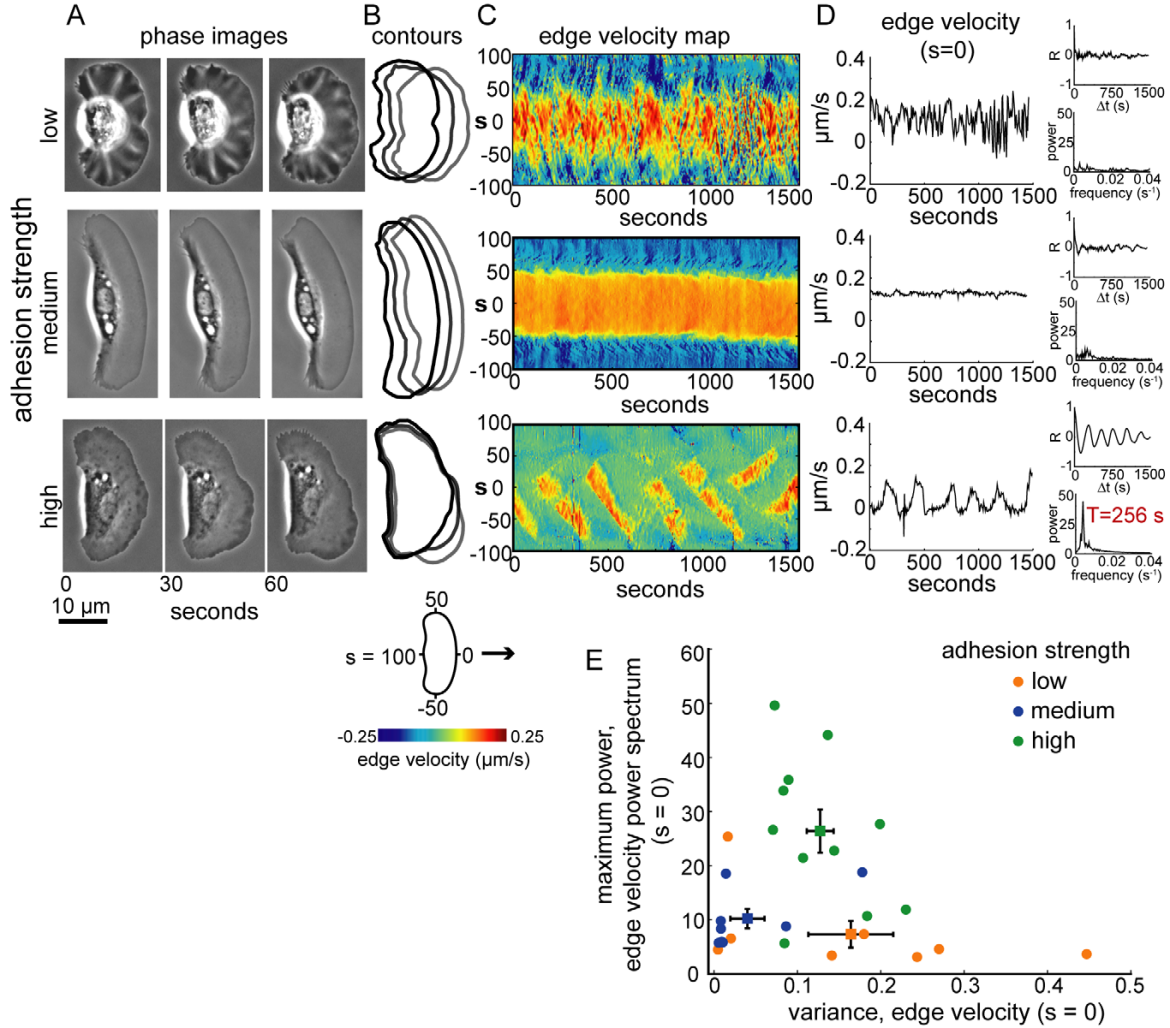
Oscillations in cell shape emerge as adhesion strength increases. (A–B) Phase contrast images (A) and cell contours (B) from representative cells crawling at low (top), intermediate (middle), and high (bottom) adhesion strength. (C) Edge velocity maps for each cell shown in (A). The velocity of the cell boundary at each point, *s*, around the cell perimeter is plotted over time. Hot colors represent protrusion of the cell boundary, and cold colors represent retraction. (D) Velocity of the cell boundary at the center of the leading edge (*s* = 0) is plotted over time. The upper inset is the autocorrelation function for the edge velocity, and the lower inset is the power spectrum of the autocorrelation function. Velocity of the leading edge of the cell plated on the high adhesion strength surface oscillated with a period of 256 seconds. (E) The variance of the edge velocity at *s* = 0 is plotted versus the maximum power in the edge velocity power spectrum for cells plated on low (n = 8), intermediate (n = 8), and high (n = 11) adhesion strength surfaces. Squares represent the average values for each population; error bars indicate standard error of the mean.

In contrast to its modest effect on cell speed, we found that the adhesion strength of the underlying surface had a dramatic effect on cell morphology (Figures 1 and 2, Movies S1–S3). To quantify the effect of adhesion strength on cell shape, we first determined the principal modes of shape variation for large populations of cells plated on low, intermediate, and high RGD densities by principal component analysis of aligned outlines of live keratocytes (Figure 1B) [34]. The major shape modes for all three populations were similar to those previously measured for keratocytes plated on untreated glass coverslips [29]: the first two modes of variation were, roughly, the projected 2D area of the cell (mode 1), and cell aspect ratio, or cell width divided by cell length (mode 2). These two modes of shape variation accounted for approximately 90% of the total variation in the low and intermediate adhesion strength populations. In the high adhesion strength population, however, the first two modes accounted for less than 80% of the total variation, and a third shape mode, left-right asymmetry, accounted for an additional 6.7% of the variation. Based on these shape modes, we measured area, aspect ratio, and left-right asymmetry directly for keratocytes plated on a range of RGD densities (Figure 1D–1F). Area and left-right asymmetry increased with adhesion strength (Figure 1D, 1F), and aspect ratio exhibited a biphasic dependence on adhesion strength, with cells crawling at intermediate adhesion strength displaying the highest aspect ratios (Figure 1E). In addition, we examined shape variability (Figure S2) and leading edge dynamics (Figure 2) in individual cells. Although cells on low adhesion strength surfaces displayed more variable protrusion and retraction rates than cells on intermediate adhesion (Figure 2C–2E), both maintained persistent symmetrical shapes over long time periods with constant area and only slightly fluctuating aspect ratio and left-right asymmetry (Figure S2). While cells on high adhesion strength surfaces also maintained constant areas (Figure S2), they exhibited traveling waves of protrusion (Figure 2C–2E). In these cells, protrusion of the leading edge oscillated with periods ranging from 100 to 400 seconds, resulting in large oscillations in aspect ratio and left-right asymmetry (Figure S2).

**Figure 3:**
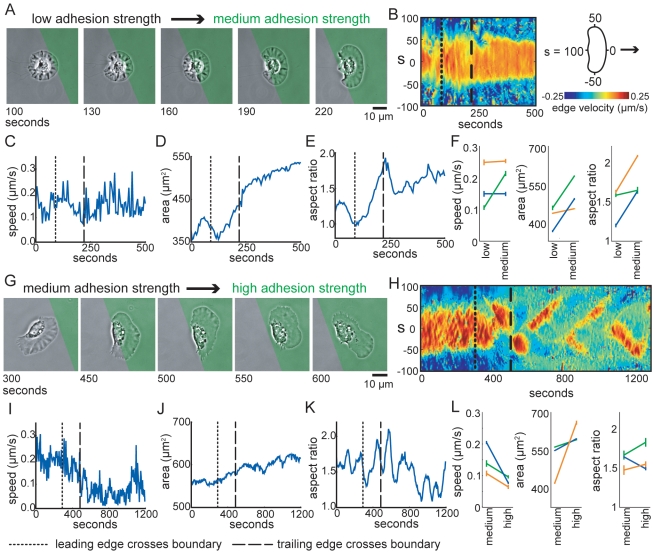
Individual cells transition between adhesion-dependent migration regimes. Cells crawling on micropatterned surfaces were imaged as they crossed boundaries between low and intermediate adhesion strength regions (A–F) and medium and high adhesion strength regions (G–L). (A,G) Phase images. The green overlay represents the region of medium (A) or high (G) adhesion strength. Edge velocity maps (B,H), cell velocity (C,I), area (D,J), and aspect ratio (E,K) are plotted over time for the cells shown in A and G. The dotted and dashed lines indicate when the leading and trailing edges crossed the boundaries, respectively. (F,L) Average cell speed, area, and aspect ratio are plotted for three cells before (left) and after (right) crawling from low to medium adhesion strength regions (F) or medium to high adhesion strength regions (L). Error bars indicate standard deviation for the individual cells.

To rule out the possibility that the observed adhesion-dependent migration behaviors were due to long-term adaptation to the different surfaces, we imaged individual cells as they crawled on micro-patterned surfaces, crossing from regions of low adhesion strength to regions of intermediate adhesion strength (Figure 3A–3F, Movie S4), and from regions of intermediate adhesion strength to regions of high adhesion strength (Figure 3G–3L, Movie S5). Area and aspect ratio increased immediately as cells crossed from low to medium adhesion strength regions (Figure 3D–3E), and speed decreased and waves of protrusion emerged as cells crossed from medium to high adhesion strength regions (Figure 3G–3I, 3L). Thus, changes in adhesion strength, in the absence of any long-term adaptation, are sufficient to switch keratocytes among three migration regimes: cells on low adhesion strength are small, round, and slow-moving; cells on intermediate adhesion strength surfaces are fan-shaped and fast-moving; and cells on high adhesion strength surfaces are large, slow-moving, and exhibit traveling waves of protrusion.

### General Model for Keratocyte Shape Determination: Actin Polymerization and Retrograde Flow Control Cell Boundary Expansion and Retraction

**Figure 4:**
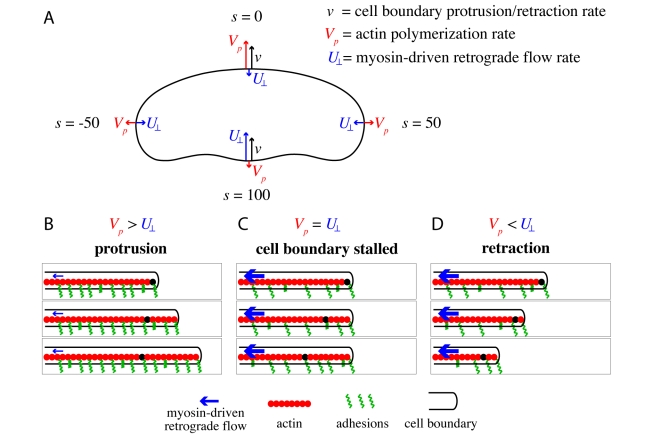
General model for keratocyte shape. (A) The expansion/retraction rate of the cell boundary is given by 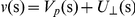, where 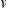 is the expansion/retraction rate, 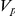 is the rate of actin polymerization, 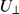 is the normal component of the centripetal bulk flow 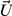 of the viscous F-actin network, and 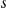 is the position along the cell boundary. In a migrating cell, the actin network, myosin, and adhesions must be organized such that 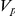 is greater than 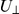 at the front of the cell (s = 0), and 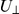 is greater than 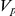 in the cell rear (s = 100). The corners of the cell are defined by the point where 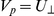 (s = ±50). (B-D) Cell-substrate adhesions (green springs) oppose myosin-driven retrograde flow (blue arrows) of the actin network (red). When adhesion is strong, or contractile forces are low, the actin network is stationary with the respect to the underlying surface (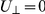) and actin polymerization drives protrusion of the cell boundary (B). When adhesion is weak or contractile forces are high (C, D), the actin network moves with respect to the underlying surface (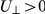). If the rate of polymerization is equal to the rate of retrograde flow (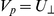) then the cell boundary is stationary (C), but if actin polymerization is less than the rate of retrograde flow (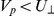) the cell boundary retracts (D).

We set out to develop a physical model for keratocyte shape determination that recapitulates the observed adhesion-dependent changes in steady-state cell shape. Our model builds on the graded radial extension model for keratocyte motility, which proposes that local expansion of the cell boundary occurs perpendicular to the cell edge, with maximal rates of extension and retraction at the center of the leading and trailing edges, respectively, and minimal rates of expansion at the cell sides [27]. These expansion and retraction rates emerge from mechanical and biochemical feedback among adhesions, the treadmilling actin network, myosin, and the inextensible membrane. Specifically, we propose that at each point along the cell perimeter, actin polymerization and myosin II-driven retrograde flow of the actin network with respect to the underlying surface add vectorially to give the effective expansion/retraction rate: 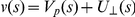, where 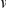 is the expansion/retraction rate, 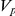 is the rate of actin polymerization, 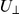 is the normal component of the centripetal bulk flow 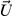 of the viscous F-actin network, and 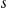 is the position along the cell boundary, with 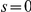 at the center of the leading edge of the cell (Figure 4A). The actin network flows inward from the cell boundary, so 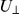 is negative. In order for a cell to migrate persistently, the molecular machinery that controls actin polymerization and retrograde flow must be organized such that actin polymerization is greater than retrograde flow in the front of the cell and retrograde flow is greater than polymerization in the rear of the cell (Figure 4B, 4D), with the cell corners set by the points where the rate of polymerization is equal to the rate of retrograde flow (Figure 4C). We propose that the rate of actin retrograde flow depends on the mechanical balance between myosin-mediated contraction of the actin network and the adhesive forces that resist retrograde flow, and that the rate of actin polymerization depends on mechanical feedback between the actin network and the cell membrane. Sub-models for actin retrograde flow and actin polymerization are presented below. This mechanical model does not evoke any specific effects of adhesion-based signaling on actin cytoskeletal dynamics; instead it treats adhesion simply as a frictional force. Nonetheless, this simple model is sufficient to recapitulate our experimental observations on cell speed, shape, and protein distributions with remarkable accuracy.

### Effects of Cell-Substrate Adhesion Strength on Actin Network Flow and Myosin Localization

The rate of retrograde actin network flow, 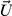, depends on the distribution of myosin-driven contractile forces, the viscosity of the actin network, and the adhesion forces that resist contraction [38]. To compute 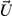, we used a model for viscous contractile flow of the lamellipodial actin network, adapted from [38]. In a simplified version of this model, the rate of the flow is determined by the balance between myosin contraction and adhesion strength (Figure 4B–4D): 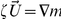 gives the local contractile forces pulling on the adhesions, where 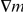 is the gradient of the myosin-generated stress and 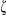 is the effective adhesion friction coefficient that quantifies the notion of the adhesion strength used to describe the experimental data. A complete model for actin network flow includes passive viscous stresses in the F-actin network (see Text S1). For simplicity, we assume that the adhesion drag coefficient 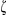 and F-actin viscosity are spatially constant and that myosin stress is isotropic and linearly proportional to the myosin density. To account for the myosin distribution, we assume that myosin binds and moves with the actin network. In the reference frame of the cell, the actin network and myosin molecules move towards the rear of the cell with velocity 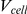, the net cell migration velocity 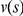 at 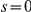, and flows inward from the cell boundary with velocity 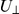.

**Figure 5:**
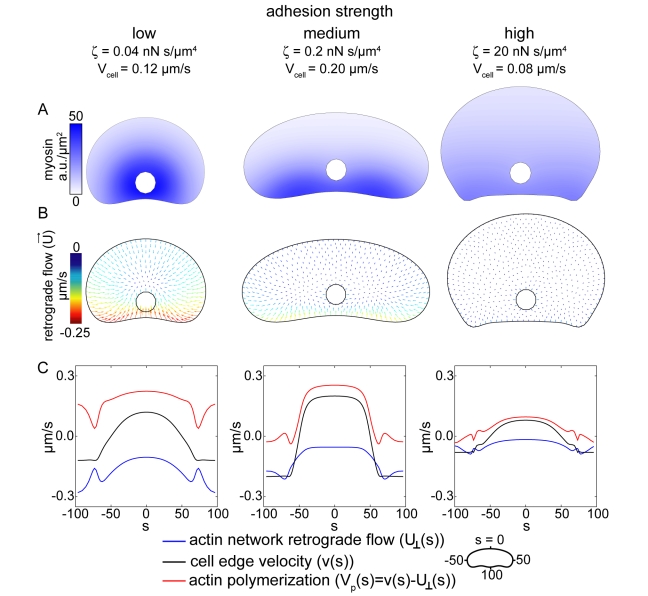
Simulated myosin and retrograde flow patterns. Coupled myosin and flow distributions were computed on the fixed cell shapes for the indicated values for the adhesion drag coefficient, ζ, and cell speed, *V_cell_*. Cell shape and *V_cell_* were taken from the experimental data. (A) Myosin distributions. (B) Actin network retrograde flow. The direction and magnitude of local actin network movement with respect to the underlying substrate is indicated by color-coded arrows; hot colors correspond to faster flow. (C) Distributions of the computed normal component of the centripetal flow around the boundary (blue), polymerization rate (red) and net protrusion/retraction rate (black). The centripetal flow rates at the boundary were taken from the flow maps shown in (B). The actin polymerization rates are the rates required to maintain the input cell shape, given the simulated retrograde flow patterns. See Text S1 for a detailed description of the model parameters.

**Figure 6:**
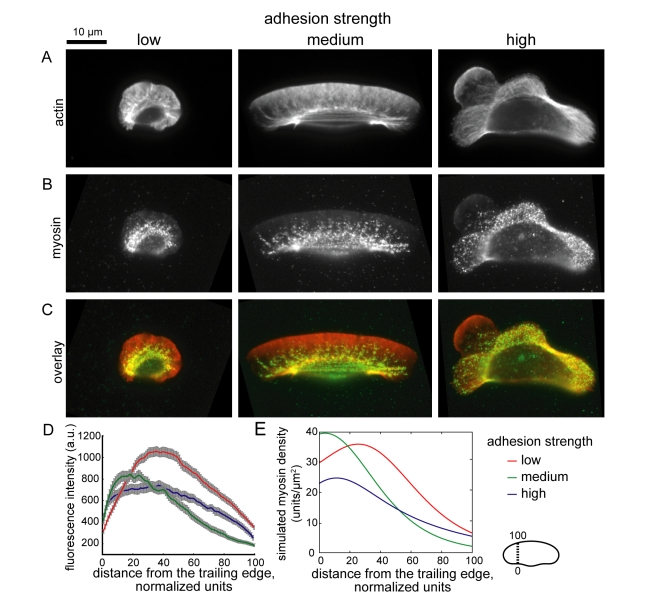
Adhesion strength affects myosin distribution patterns. Images of cells plated on low (left), intermediate (center), and high (right) adhesion strength surfaces and labeled for actin with fluorescent phalloidin (A) and immunolabeled for myosin (B). (C) Overlays of the actin and myosin images; actin is pseudo-colored red, and myosin is pseudo-colored green. (D,E) Experimental (D) and simulated (E) myosin distributions, measured from the cell rear (point 0) to the cell front (point 100) on either side of the cell body, for cells plated on low (n = 31 cells), intermediate (n = 22 cells), or high (n = 20 cells) adhesion strength surfaces. Error bars indicate standard error of the mean.

To determine the effect of adhesion strength on 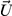, we simulated myosin distribution and retrograde flow patterns using low, intermediate, and high values for the adhesion drag coefficent, 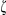, and input cell shapes and cell velocities measured from the experimental data (Figure 5, see Text S1). Our simulations predict adhesion-dependent feedback between myosin localization and actin network flow. The magnitude of retrograde actin network flow increases as 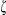 decreases (Figure 5B–5C) and this increase in retrograde flow has consequences for the myosin localization pattern. At intermediate and high 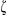, the actin network is nearly stationary with respect to the underlying substrate, so myosin localization in the cell coordinate system is largely controlled by 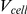: actin-bound myosin moves towards the cell rear as the cell moves forward at velocity 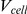. Cells crawling at high adhesion strength exhibit significantly slower velocities than cells crawling at intermediate adhesion strength (Figure 1C), and our simulations predict that the measured difference in cell speed should result in different myosin distribution patterns, with increased enrichment of myosin in the cell rear in fast-moving cells crawling at intermediate adhesion strength compared to slow-moving cells crawling at high adhesion strength (Figure 5A, center and right panels; Figure 6E). At low 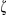, in addition to moving towards the cell rear in the cell coordinate system with the rate 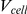, the actin network moves with respect to the underlying substrate, flowing inward from the cell boundary with the rate 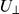 and resulting in the accumulation of actin-bound myosin in a ring around the cell body (Figure 5A, left panel). The myosin distribution pattern, in turn, affects the pattern of retrograde flow, with the highest rates of flow occurring in regions where myosin and its density gradients are enriched (Figure 5A, 5B). Although these retrograde flow and myosin distribution patterns were computed on the average cell shapes measured from experimental data, similar patterns were observed when the same input shape was used for all three cases (Figures S3 and S4), indicating that the observed differences arise from the variations in adhesion strength and are not solely due to differences in the input cell shape.

This sub-model for myosin-mediated retrograde flow makes quantitative predictions about myosin localization and retrograde flow in cells on different adhesion strength surfaces. First, the model simulations predict specific, adhesion-dependent myosin localization patterns (Figure 5A). To test this, we examined myosin localization patterns in cells plated on low, intermediate, and high adhesion strength surfaces (Figure 6). As predicted, myosin localized to a ring around the cell body in cells crawling at low adhesion strength (Figure 6B, left panel) and localized to the cell rear in cells crawling at intermediate adhesion strength (Figure 6B, center panel). In cells crawling at high adhesion strength, myosin was largely excluded from protruding portions of the leading edge and under the cell body, but was more uniformly distributed in the rest of the cell (Figure 6B, right panel). We compared these experimental myosin distributions to the model simulations by measuring average myosin densities from the cell front to the cell rear in populations of cells (Figure 6D). We found that myosin accumulated in the cell rear at intermediate adhesion strengths but was more uniformly localized in cells at high adhesion strengths, consistent with the model simulations (Figure 6E). We also found that peak myosin densities were shifted towards the cell interior in cells crawling at low adhesion strengths, consistent with increased inward flow of the actin network and attached myosin molecules at low adhesion strengths.

**Figure 7:**
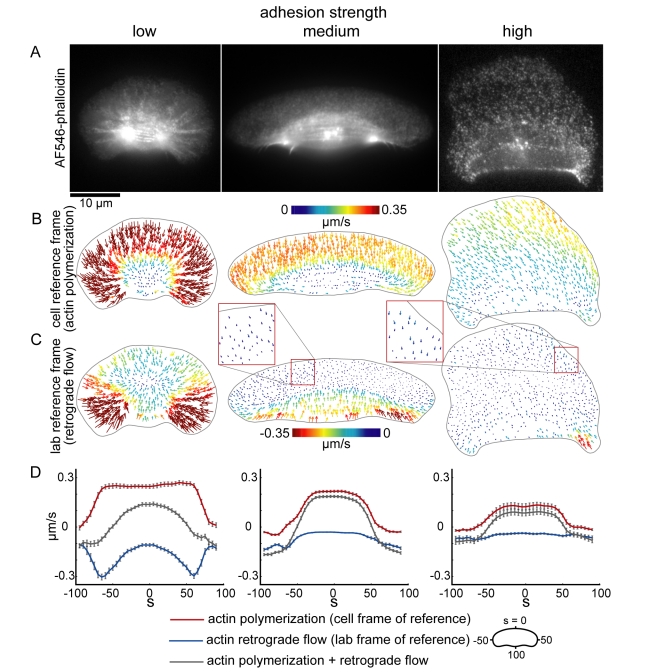
Actin polymerization and retrograde flow rates decrease as adhesion strength increases. (A) Images of keratocytes labeled with a low concentration of AlexaFluor546-phalloidin, plated on low (left), intermediate (center), and high (right) adhesion strength surfaces. (B,C) Actin network flow maps in the cell frame of reference (B), corresponding to actin polymerization, and in the lab frame of reference (C), corresponding to retrograde flow of the actin network, are shown for the cells shown in (A). (D) Average actin polymerization rates (red lines), actin retrograde flow rates (blue lines) measured in populations of cells plated on low (n = 36 cells), intermediate (n = 46 cells), and high (n = 25 cells) adhesion strength surfaces are plotted for each point around the cell perimeter. The gray lines are the effective cell boundary expansion/retraction rates calculated by adding the measured actin polymerization and retrograde flow rates. Error bars indicate standard error of the mean.

Second, the model simulations predict that retrograde flow should increase as adhesion strength decreases, with the fastest flow localized to the rear of the cell where myosin is enriched (Figure 5). To test this, we measured actin network dynamics by fluorescence speckle microscopy (Figure 7). We measured retrograde flow of the actin network by measuring flow relative to the underlying substrate (the lab frame of reference, Figure 7C) as well as actin polymerization rates by measuring flow relative to the cell perimeter (the cell frame of reference, Figure 7B). As predicted, retrograde flow of actin increased as adhesion strength decreased (Figure 7C, 7D). The rate of actin polymerization also increased with decreasing adhesion strength (Figure 7B and 7D), suggesting that in addition to affecting retrograde flow, adhesion strength also affects actin network growth rates. Moreover, the distribution of actin polymerization and retrograde flow rates around the cell boundary varied with adhesion strength (Figure 7D). At low adhesion strengths, the magnitude of retrograde flow was graded (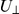 increased from 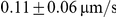 at 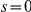 to 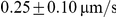 at 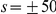) and the magnitude of actin polymerization along the leading edge was constant (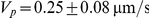 at 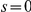 and at 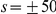, n = 35 cells). At intermediate and high adhesion strengths, in contrast, the magnitude of retrograde flow was nearly constant along the leading edge (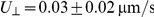 at 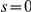 and 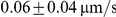 at intermediate adhesion strength; 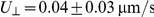 at 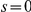 and 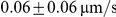 at 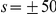 at high adhesion strength) and the rate of actin polymerization decreased in a dramatic, step-like fashion (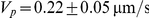 at 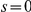 and 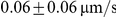 at 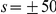 at intermediate adhesion strength, n = 46 cells; 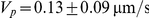 at 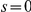 and 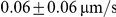 at 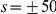 at high adhesion strength, n = 23 cells). These measured actin polymerization and retrograde flow distributions are consistent with the simulated distributions (compare Figures 5C and 7D) and suggest that as adhesion strength increases, the mechanism for cell shape and speed determination switches from dependence on retrograde actin network flow to dependence on actin polymerization. Specifically, these results suggest that cells at low adhesion strength are rounder and slower than cells at intermediate adhesion strength, despite increased actin polymerization, because of increased retrograde flow at the cell front and sides, whereas cells on high adhesion strength are slower and rounder because of reduced actin polymerization rates.

**Figure 8:**
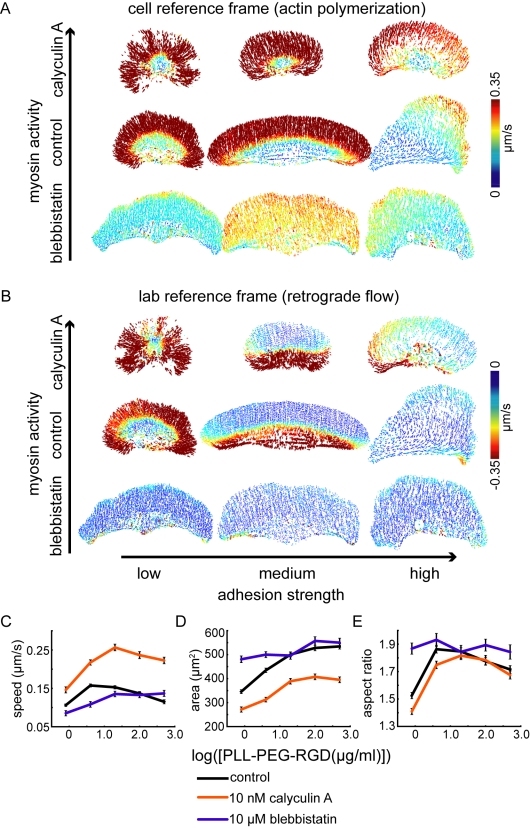
The balance between adhesion strength and myosin activity determines actin polymerization and retrograde flow rates and cell speed and shape. (A,B) Representative flow maps in the cell frame of reference, corresponding to actin polymerization (A), and lab frame of reference, corresponding to retrograde flow (B), are shown for cells crawling at low (left), intermediate (center), and high (right) adhesion strength and treated with either 10 nM calyculin A (top), 10 µM blebbistatin (bottom), or no drug (middle). Average cell speed (C), area (D), and aspect ratio (E) for populations of cells treated with blebbistatin (blue lines) or calyculin A (orange lines) are shown for cells plated on surfaces coated with the indicated PLL-PEG-RGD concentrations. The data for control cells, shown in Figure 1, is re-plotted here for comparison (black lines). Error bars indicate standard error of the mean.

Finally, the model predicts that tuning the level of myosin activity with respect to adhesion strength should affect actin retrograde flow rates, with fast retrograde flow occurring when myosin activity is high relative to adhesion strength (Figure S5). To test this prediction experimentally, we treated cells crawling at low, intermediate, and high adhesion strengths with either blebbistatin, a myosin II inhibitor [39], or calyculin A, a phosphatase inhibitor that activates myosin contraction [40]. Although calyculin A has multiple targets, its effects on cell shape and speed in keratocytes appear to be dominated by its effects on myosin II activity (see Figure S6 and Text S2). As predicted, we found that myosin inhibition reduced retrograde flow and activation of myosin contraction increased retrograde flow (Figure 8B, Figure S7), consistent with model simulations (Figure S5). Actin polymerization rates also increased with increasing myosin activity (Figure 8A, Figure S7), indicating that the balance between adhesion strength and myosin contraction (rather than the absolute magnitude of either) determines the rate of actin network growth, as well as the rate of retrograde flow. In addition, we found that myosin-dependent changes in actin polymerization and retrograde flow rates were associated with changes in cell shape and velocity (Figure 8C–8E). Area and aspect ratio increased in cells treated with blebbistatin, particularly in cells at low adhesion strength, and decreased in cells treated with calyculin A (Figure 8D–8E). Cell velocity, conversely, increased in cells treated with calyculin A, at all adhesion strengths, suggesting that increased actin polymerization more than compensates for increased retrograde flow when myosin activity is enhanced.

### Establishment of Adhesion and Actin Network Density Distributions

The sub-model for myosin-driven retrograde flow detailed above is consistent with experimental measurements of retrograde flow and cell shape, but by itself does not address the observed adhesion- and myosin-dependent actin polymerization distributions (Figures 7B and 8A). Previously published measurements of keratocyte shapes and actin network distributions are consistent with a model in which the rate of actin polymerization along the cell perimeter, 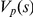, emerges from mechanical feedback between the actin network and the cell membrane [29]. Specifically, 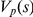 is thought to depend on the organization and density of actin filaments, the concentration of free actin monomers, and membrane tension, which imposes a force on the actin network, slowing filament growth [29]: 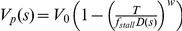, where 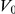 is the free actin polymerization rate proportional to the actin monomer concentration, 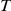 is membrane tension, 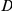 is the local density of actin filaments, 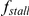 is the force at which polymerization stalls, and *w* ∼ 8 is the parameter characterizing the force-velocity relationship for actin polymerization. We assume that within individual cells actin monomer concentration and membrane tension are globally constant—actin monomers rapidly diffuse throughout the cell, and local perturbations in membrane tension rapidly equilibrate—but actin filament densities along the leading edge have been shown to be graded in keratocytes, with enrichment of branched filaments at the center of the leading edge and enrichment of bundled filaments at the trailing edge [29],[30]. Large adhesion complexes localize to the trailing edge in keratocytes [30],[41],[42] and large, stable adhesions are associated with reduced protrusion in Chinese hamster ovary (CHO) cells [43]. Myosin also localizes predominantly to the cell rear in keratocytes (Figure 6) and is thought to promote actin depolymerization [26]. Therefore, we propose that antagonism between adhesion complexes and the dendritically branched actin network and myosin-mediated actin depolymerization determine the actin filament distribution along the cell perimeter, 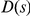 (see Text S1).

**Figure 9:**
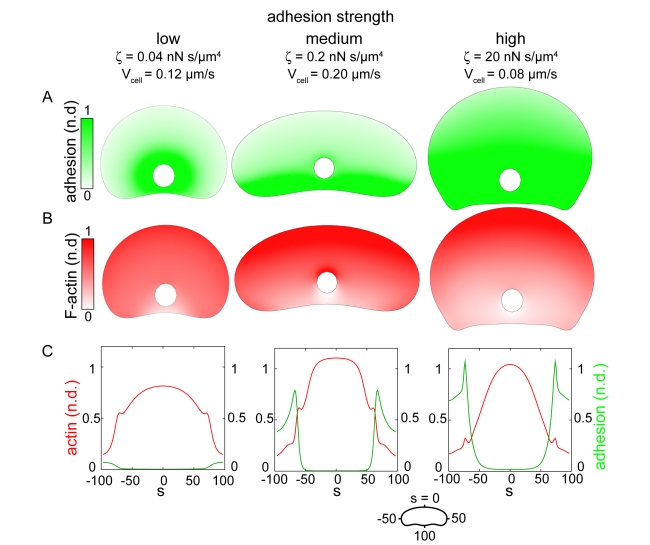
Simulated adhesion and actin filament distribution patterns. Coupled adhesions and actin distributions were computed from the actin network flow patterns shown in Figure 5 for low (left), medium (center) and high (right) adhesion strengths. (A) Simulated adhesion distributions. (B) Simulated F-actin distributions. (C) Distributions of the computed adhesion (green) and F-actin (red) densities around the cell perimeter. Units are non-dimensionalized (n.d.). See Text S1 for simulation parameters.

**Figure 10:**
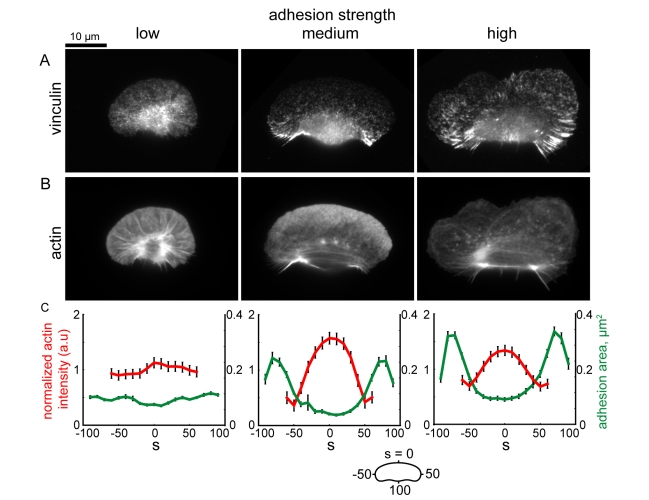
Large, elongated adhesions spatially correlate with a reduction in branched actin network density. (A,B) Images of cells immunolabeled for vinculin (A) and labeled for actin with fluorescent phalloidin (B). (C) Average actin intensity (red line) and adhesion area (green line) are plotted for points along the cell perimeter for cells plated on low (n = 176 cells), medium (n = 136 cells), and high (n = 179 cells) adhesion strength surfaces. Actin intensities are normalized such that the mean intensity for each cell is equal to 1. Error bars indicate standard error of the mean.

We simulated adhesion and actin distributions and the rate of actin polymerization along the cell boundary, 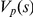, based on the retrograde flow maps and myosin distributions simulated in Figure 5. To model the adhesion distribution (which we model as a continuous distribution, rather than as discrete adhesion complexes), we assume that adhesions assemble and disassemble with constant rates throughout the cell. Adhesions are malleable structures that are partially dragged along the surface due to coupling with the actin network [11]; thus, in the cell coordinate system, their localization depends on the speed with which the cell moves over them, and the rate of retrograde flow of the actin network within the cell. Simulations of this model (see Text S1 for a discussion of model parameters) show that at low adhesion strength, adhesions are swept inward due to high rates of retrograde flow (Figure 9A, 9C, left), and therefore do not inhibit polymerization of the branched actin network, resulting in uniform distributions of actin filaments around the cell perimeter (Figure 9B–9C, left). In contrast, at intermediate and high adhesion strengths, reduced rates of retrograde flow allow adhesion complexes to accumulate in the cell rear (Figure 9A, 9C, center and right) and inhibit actin polymerization, resulting in enrichment of actin filaments at the center of the leading edge (Figure 9B, 9C, center and right). We examined actin network organization and adhesion distribution (as measured by vinculin localization) in cells plated on low, intermediate, and high adhesion strength surfaces (Figure 10), and found that adhesion complexes were most enriched in regions of the cell predicted to have the highest adhesion densities (compare the simulated distributions in Figure 9A and 9C with the experimental data shown in Figure 10A and 10C). Small, punctate adhesions localized to a ring around the cell body in cells plated at low adhesion strengths (Figure 10B, left panel), whereas larger, elongated adhesions localized to the trailing edge in cells at intermediate adhesion strength (Figure 10B, center panel). At high adhesion strength, large adhesions were biased towards the cell rear, but also localized to stalled portions of the leading edge (Figure 10B, right panel). In all cases, elongated adhesions were spatially correlated with a reduction in dendritically branched F-actin and an increase in bundled actin filaments, resulting in uniform actin distributions in cells plated on low adhesion strength surfaces, and in peaked distributions in cells on intermediate and high adhesion strength surfaces (Figure 10C, compare to Figure 9C). Overall, these results are consistent with the hypothesis that large adhesions antagonize the assembly of a dendritically branched actin network.

The simulated and experimentally observed distributions of mature adhesions described above are spatially heterogeneous (Figures 9 and 10), which is seemingly at odds with our initial assumption that the adhesion drag coefficient 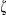 is spatially constant. However, the relationship between adhesion size and age and adhesion strength is not well understood: depending on the cell type, traction force measurements suggest that adhesion strength either increases [22], decreases [44], or does not correlate [10] with adhesion size. Therefore, we simulated actin network flow patterns and myosin, adhesion, and actin distributions for three different cases. In the cases where 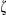 is spatially constant (Figures 5 and 9) or decreases with adhesion density (Figure S8; see Text S1), the simulated patterns matched the experimentally observed patterns. However, in the case where 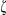 increases with adhesion density, the simulated patterns of retrograde actin flow did not match the experimentally observed patterns (Figure S9). Specifically, the rate of retrograde flow was nearly constant around the cell perimeter at intermediate values for 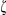 rather than increasing in the cell rear as observed experimentally (Figure S9C, S9D, Figure 7C, 7D). Thus, our findings are most consistent with a pattern of adhesion strength that is nearly constant or decreasing from the front to the rear of migrating cells. Indeed, this latter pattern is most consistent with the idea that cells must release adhesions in the rear in order to move forward, and is also well-supported by recent experiments that measured the spatial pattern of traction forces exerted on the underlying substrate by migrating keratocytes [9].

### Emergence of Cell Shape from the Interaction of Mechanical Forces

**Figure 11:**
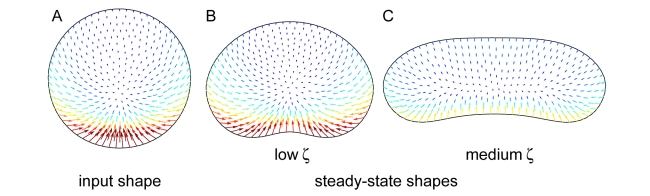
Dynamical simulations of cell shape for different adhesion drag coefficents recapitulate experimentally observed differences in cell shape. Cell shape and actin network flow were simulated using an iteration procedure (see Text S1). Cell shape and actin flow at the cell boundary are shown for the input shape (A), the stable shape that evolved at low adhesion strength (B; ζ = 0.04 nNs/µm^4^), and the stable shape that evolved at intermediate adhesion strength (C; ζ  = 0.2 nNs/µm^4^).

Our proposed model nicely recapitulates experimental actin flow patterns, as well as myosin, adhesion, and actin distributions, for low, intermediate, and high adhesion strengths. Finally, to model the effect of adhesion strength on cell shape, we performed dynamic modeling of actin polymerization, retrograde flow, and cell shape using an iterative procedure (see Text S1, Movie S6). Starting with a round input shape (Figure 11A), we solved for 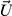 and 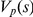 using a low adhesion drag coefficient, 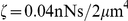 (see Text S1). We allowed the input shape to deform in response to the simulated expansion and retraction rates, and the simulation was repeated until the shape converged to a stable shape and flow pattern. The simulations converged to a shape with low aspect ratio and fast retrograde flow at the cell sides and rear (Figure 11B), consistent with the experimental data (Figure 1E, Figure 7C, left panel). Then, we increased the adhesion drag coefficient to an intermediate value, 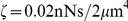, and repeated the simulations again until the shape converged to a new stable shape. With the intermediate adhesion drag coefficient, the simulations converged to a shape with higher aspect ratio and lower retrograde flow rates (Figure 11C), also consistent with the experimental data (Figure 1E, Figure 7C, center panel). Altogether, these results demonstrate that a self-consistent, self-organizing model in which cell shape emerges from interactions among the actin network, myosin, adhesions, and the cell membrane recapitulates the observed adhesion-dependent changes in steady-state cell shape.

## Discussion

We have presented a model in which overall cell shape depends on mechanical feedback among the actin network, myosin, adhesions, and the cell membrane. In this model, extension of the cell perimeter, and thus cell shape, is determined by the vectoral sum of retrograde flow of the actin network and actin network growth. Specifically, the cell front is defined by the region where polymerization rates are greater than retrograde flow rates, the cell rear is defined by the region where retrograde flow rates are greater than actin polymerization rates, and the relative positions of the cell corners are defined by the points where the two rates are perfectly balanced (Figure 4). The rate of retrograde flow at each point around the cell perimeter depends on the local balance between adhesion and myosin-mediated contraction of the actin network. The spatial pattern of retrograde flow rates is determined by an asymmetric distribution of myosin molecules: myosin associates with the actin network and accumulates in the rear of the cell as the cell moves forward, resulting in increased myosin contraction and retrograde flow in the cell rear. The rate of actin polymerization at each point around the cell perimeter depends on the density of branched actin filaments. Adhesions, like myosin, accumulate in the cell rear as the cell moves forward, and we propose that myosin- and adhesion-mediated reduction of branched actin density in the cell rear effectively biases fast actin polymerization towards the cell front.

Simulations of this model predict an adhesion-dependent switch between mechanisms that determine cell shape. At intermediate and high adhesion strengths, myosin and adhesions localized to the trailing edge reduce the density of the branched actin network, thereby establishing a graded distribution of pushing actin filaments (Figures 5A and 9). As the density of actin filaments decreases towards the cell sides, membrane tension stalls actin polymerization [29]. Retrograde flow of the actin network is slow and fairly constant from the cell front to the cell sides (Figure 5B, 5C), so reduced actin polymerization, rather than increased retrograde flow, sets the relative positions of the cell corners. At low adhesion strength, in contrast, myosin and adhesions are swept to the cell interior by retrograde flow of the actin network (Figure 5A and 9A), preventing myosin- and adhesion-mediated reduction of F-actin density and resulting in a uniform distribution of F-actin and actin polymerization rates from the cell front to the cell sides (Figure 5C and 9C). Retrograde flow, on the other hand, is graded, increasing dramatically from the cell front to the cell side (Figure 5B, 5C). Thus, at low adhesion strength, increased retrograde flow, rather than reduced actin polymerization, sets the relative positions of the cell corners. We have presented experimental evidence that is consistent with the predictions of this model: as adhesion strength decreased, retrograde flow of the actin network increased (Figure 7C, 7D), myosin and adhesions switched from localization to the trailing edge to localization to a ring around the cell body (Figures 6 and 10B, 10C), and the actin filament density along the leading edge became less graded (Figure 10A, 10C). In addition, we found that inhibiting or activating myosin contraction either decreased or increased retrograde flow (Figures 8, S5), as predicted by model simulations (Figure S4). Finally, numerical simulations correctly recapitulated the effect of adhesion strength: as predicted (Figure 11), keratocytes were fan-shaped at intermediate adhesion strength and round at low adhesion strength (Figure 1E). These results are consistent with our model in which the self-organization of the dynamic actin network, myosin, and adhesions determines motile cell shape.

Integrin-based adhesions are both mechanical structures that resist myosin-driven actin network flow and organizing centers that localize biochemical signals that contribute to the organization of the actin network [13],[45]. The mechanical model for cell shape determination described here does not explicitly incorporate biochemical signaling pathways. However, we argue that the myriad effects of integrin signaling are relevant for cell shape determination only insofar as they affect the localization and activity of molecules that contribute to mechanical feedback among the actin network, adhesions, myosin, and the cell membrane. For example, integrin signaling is thought to promote both Rac and RhoA GTPase activity, with nascent adhesions promoting Rac activity [14] and mature adhesions promoting RhoA activity [19]. Increased Rac activity promotes protrusion by activating Arp2/3 [15],[16], increasing the number of barbed ends. Therefore, the effect of increased Rac activity can be reduced to the mechanical consequences of increasing the density of actin filaments at the cell boundary, which is described in our mechanical sub-model for actin polymerization. One of the consequences of increased RhoA activity, on the other hand, is activation of myosin light chain kinase and up-regulation of myosin activity [21], which can be reduced to the mechanical consequences of increasing contractile forces, which is described in our mechanical model for actin network retrograde flow. Recent efforts to model individual mechanical process important for cell migration have included some aspects of integrin signaling, including a recent kinetic model for actin-integrin “clutch” dynamics [46]. Although our model does not currently incorporate integrin signaling, future efforts could be made to include the specific contributions of signaling pathways to the general mechanical mechanisms described here.

In our model for actin network polymerization, antagonism between adhesions and the branched actin network, along with myosin-mediated actin depolymerization and cooperative actin network branching, establishes a graded distribution of F-actin along the leading edge. This graded actin network distribution has been measured previously [29],[30]; here we show that decreased actin filament densities spatially correlate with large, elongated adhesions at intermediate and high adhesion strengths. At low adhesion strength, keratocytes exhibit only small, punctate adhesions and have relatively uniform F-actin distributions along the leading edge. These results are consistent with the idea that mature adhesions inhibit assembly of the branched actin network, but the mechanism for this antagonism between mature adhesions and the branched actin network is unknown. Mature adhesions have been shown to promote formation and polymerization of actin bundles [47]–[49] and this adhesion-dependent filament bundling may be sufficient to switch the actin network to a bundled architecture incompatible with lamellipodia-based protrusion. In addition, nascent adhesions are thought to trigger a positive feedback loop that promotes actin polymerization and leading edge protrusion downstream from Rac GTPase [14], and adhesion maturation may antagonize actin polymerization by disrupting this feedback loop [43].

Our model recapitulates the observed adhesion-dependent changes in retrograde flow patterns and myosin, adhesion, and F-actin distributions, but it does not directly account for adhesion- and myosin-dependent changes in actin polymerization rates. We have found that actin polymerization rates increase with decreasing adhesion (Figure 7) and decrease with decreasing myosin activity (Figure 8, Figure S5). Actin polymerization rates depend on the concentration of free actin monomers and the force per actin filament imposed by the cell membrane [50],[51]. Membrane tension may increase as the strength of adhesion increases relative to myosin contraction: when adhesion strength is high and myosin activity is low, cell area increases, potentially stretching the cell membrane and increasing membrane tension. However, measurements of membrane tension in keratocytes at low, intermediate, and high adhesion strengths have shown that changes in membrane tension alone are not sufficient to account for the changes in actin polymerization rates (E. Barnhart, A. Leibler, and K. Keren; unpublished data). The balance between adhesion and myosin strength may also affect the concentration of actin monomers: adhesions have been shown to promote polymerization of actin bundles [47] and myosin contraction has been shown to promote actin depolymerization [26]. Therefore, we propose that the balance between myosin contraction and adhesion strength influences the concentration of free actin monomers, with the monomer concentration increasing as adhesion size decreases or myosin activity increases, resulting in a global increase in the actin polymerization rate.

Our results indicate that increased actin polymerization can compensate for increased actin retrograde flow in keratocytes. We found that cell speed increased in cells treated with calyculin A, a phosphatase inhibitor that promotes myosin contraction [40], at all adhesion strengths (Figure 8C). In these cells, increased actin polymerization more than compensated for increased retrograde flow of the actin network at the front of the cell (Figure S5). Cell speed did not change in some individual cells as they crawled from regions of low to intermediate adhesion strength (Figure 3C, 3F), consistent with the idea that increased actin polymerization compensates for increased retrograde flow at low adhesion strengths. Similarly, non-adhesive dendritic cells have been shown to migrate at speeds comparable to those of adhesive dendritic cells despite increased retrograde flow, suggesting that increased actin polymerization compensates for increased retrograde flow to maintain constant cell speed [52]. Together, these results are consistent with the idea that cell speed is determined by the sum of actin polymerization and retrograde flow rates in simple-shaped, fast-moving cells like keratocytes and dendritic cells. In slow-moving cells such as PtK1 cells, however, the relationship between actin polymerization and retrograde flow rates and cell speed is more complicated [3]: although instantaneous protrusion and retrograde flow rates in migrating PtK1 cells are comparable to those measured in keratocytes, with protrusion rates as fast as ∼0.3 µm/s and retrograde flow rates on the order of 0.01 µm/s, cell speed is an order of magnitude lower in PtK1 cells (∼0.01 µm/s in PtK1 cells, compared to 0.1 µm/s in keratocytes). The balance between adhesion strength and myosin contraction has been shown to control cell speed in these cells [3]. Unlike keratocytes, PtK1 cells do not exhibit steady-state shapes and speeds, and thus more complicated adhesion- and myosin-dependent mechanisms are likely to control cell speed in these cells.

Although fish keratocytes are renowned for their ability to maintain steady-state shapes and velocities over hundreds of microns of migration [1],[27],[29],[30],[33], they have also been shown previously to exhibit non-steady-state dynamic behaviors, including spontaneous symmetry breaking and motility initiation [53] and oscillatory retraction of the trailing edge [54]. Here we have found that traveling waves of protrusion emerge in keratocytes as adhesion strength increases (Figures 2 and 3). In these cells, similar to PtK1 cells, overall cell speed is significantly slower than protrusion rates along short sections of the leading edge (cell speed  = 0.06 µm/s, compared to maximum protrusion velocity  = ∼0.2 µm/s for the cell shown in Figure 2). The steady-state model described here cannot account for these protrusion waves, but regular oscillations in protrusion have been previously observed in other cell types [55]–[62]. Proposed mechanisms for these protrusion oscillations include mechanical feedback between adhesion formation at the leading edge and myosin contraction [58] and a mechanism in which Rho GTPase activation at the leading edge promotes initial protrusion [60]. We favor a third mechanism, in which mature focal adhesions titrate actin polymerization activators away from the leading edge, resulting in the emergence of waves of protrusion. A detailed model for oscillation of the leading edge in keratocytes crawling at high adhesion strength will be published elsewhere.

Migrating cells take many shapes and move at different velocities, ranging from triangular-shaped, slow-moving fibroblasts to amoeboid-shaped, fast-moving neutrophils and fan-shaped, fast-moving fish keratocytes [28]. These various cell shapes and migration speeds are the manifestation of the underlying dynamics and mechanics of the cytoskeleton. Top-down modeling of cell shape has demonstrated that quantitative changes in “control parameters” such as adhesion strength are sufficient to switch cells between different shapes [63]. Here, we have presented a bottom-up model for keratocyte shape determination that emerges from known biochemical and mechanical interactions among the cellular components involved in force generation for cell motility. We have found that changes in adhesion strength and myosin activity are sufficient to switch keratocytes between migration regimes, including one on high adhesion strength surfaces in which the typically fan-shaped, fast-moving keratocytes begin to resemble slow-moving fibroblasts. Thus, quantitative, rather than qualitative, differences in control parameters are likely to be sufficient to explain the different cell shapes and behaviors observed for different cell types.

## Materials and Methods

### Keratocyte Culture and Labeling

Keratocytes were cultured from the scales of the Central American cichlid *Hypsophrys nicaraguensis* as described [30]. Briefly, scales were sandwiched between two acid-washed coverslips and cultured in Leibovitz's Media (L-15) supplemented with 14.2 mM HEPES pH 7.4, 10% FBS, and 1% antibiotic-antimycotic at room temperature for 12–24 hours. Keratocytes were replated by trypsinization: cells were washed briefly with PBS and then treated with 0.1% trypsin and 1 mM EDTA in PBS for 5 minutes. The trypsin was quenched with a ten-fold excess of culture media and the cells were transferred directly to PLL-PEG-RGD coated surfaces and allowed to recover for one hour. Pharmacological agents including blebbistatin and calyculin A were applied to cells 10–30 minutes prior to imaging.

AlexaFluor546 phalloidin (AF546-phalloidin, Invitrogen) was used to label F-actin for fluorescent speckle microscopy (FSM). 2 µM AF546-phalloidin was mixed with 7 µM dATP, 7 µM GTP, and 5 µM CTP for 15 minutes at room temperature to prevent phalloidin aggregation. The phalloidin mixture was introduced into keratocytes using a small volume electroporator for adherent cells with three pulses at 150 V.

### Synthesis of Surface Coating Materials

PLL-PEG, PLL-PEG-RGD, and PLL-PEG-FITC copolymers were synthesized as described [35],[64]. Functionalized PEGs-hetero-bifunctional 3.4 kDa PEG with vinyl-sulfone (VS) and N-hydroysuccinimide (NHS) termini (VS-PEG-NHS), monofunctional 2 kDa mPEG-NHS, and 5 kDa FITC-PEG-NHS—and 15-30 kDa PLL were dissolved separately in HEPES pH 8.4, mixed at a ratio of one PEG chain per 3.5 lysine residues and final PEG concentration of 24 mM, and then stirred for 3 hours at room temperature. For PLL-PEG and PLL-PEG-FITC, all PEG chains were mPEG-NHS or FITC-PEG-NHS, respectively. For PLL-PEG-RGD, 50% of the PEG chains were VS-terminated and, after the 3 hour incubation with PLL, a 4-fold molar excess of RGD peptides (N-acetyl-GCRGYGRGDSPG-amide) was added to the reaction, which was then stirred for an additional 24 hours at room temperature before quenching with 50 mM beta-mercaptoethanol. The samples were dialyzed against ddH_2_O, lyophilized, and stored at −20°C. The PLL-PEG-FITC reaction was kept in the dark throughout the synthesis.

### Surface Preparation

To generate surfaces with a range of RGD peptide densities, PLL-PEG and PLL-PEG-RGD were dissolved in PBS and mixed at various ratios, with a final total concentration of 0.5 mg/ml. Glass coverslips were washed with acetone and isopropanol, coated with PLL-PEG/PLL-PEG-RGD for 20 minutes at room temperature with slow rocking, and thoroughly rinsed with ddH_2_O. The surfaces were either used immediately or stored for up to 24 hours at +4°C.

Surfaces patterned with 50 µm stripes were generated by microcontact printing [65]. To generate stamp masters, silicon wafers were spin-coated with photoresist (SPR 220-7.0) for 1 minute at 1700 rpm, resulting in a resist thickness of 10 µm. The resist was baked for 90 seconds at 115 C, exposed through a photomask for 45 seconds, and developed for 5 minutes in MF319 developer. Poly(dimethyl)siloxide (PDMS, Sylgard 184) stamps were prepared by mixing the resin and curing agent at a 10:1 v/v ratio. The elastomer was poured over the stamp master and degassed before baking at 80°C for 2 hours. The stamps were cleaned with acetone and isopropanol and incubated with 0.5 mg/ml PLL-PEG/PLL-PEG-RGD, plus 1% PLL-PEG-FITC, for 40 minutes in the dark. The stamps were then dried with clean air and placed on acetone-cleaned glass coverslips for approximately 15 seconds. The surfaces were washed thoroughly with ddH_2_O, backfilled with 0.5 mg/ml PLL-PEG/PLL-PEG-RGD for 20 minutes, and washed again with ddH_2_O. In all cases, the ratio of PLL-PEG-RGD to PLL-PEG was greater in the stamped solution than in the backfill.

### Immunofluorescence

Indirect immunofluorescence was performed using monoclonal mouse anti-vinculin (hVIN-1, ab11194, Abcam, Cambridge MA) and polyclonal rabbit anti-myosin antibodies (ab2480, Abcam, Cambridge MA). For vinculin staining, cells were fixed at room temperature with 4% formaldehyde in 0.32 M sucrose in PBS for 15 minutes, permeabilized with 0.5% Triton X-100 for 10 minutes, and blocked with PBS-BT (3% BSA, 0.1% Triton X-100, and 0.02% sodium azide in PBS) for 30 minutes prior to incubation with primary antibody diluted in PBS-BT. F-actin was labeled with fluorescently conjugated phalloidin. For myosin staining, cells were extracted with 4% PEG and 1% Triton X-100 in cytoskeleton stabilizing buffer (50 mM imidazole, 50 mM KCl, 0.5 mM MgCl_2_, 1 mM EDTA, 1 mM EGTA, and 0.5 M TMR-phalloidin) for 5 minutes as described [26], rinsed three times with PBS, blocked with PBS-BT for 5 minutes, and then incubated with primary antibody diluted in PBT-BT. Cells were then fixed with 4% formaldehyde in PBS for 10 minutes.

### Microscopy

Live cells were imaged on an inverted microscope (Diaphot-300, Nikon) using a 40× NA 1.3 oil Fluor or a 60× NA 1.4 oil plan-Apo objective (Nikon). Fixed cells were imaged on an upright microscope (Axioplan 2; Carl Zeiss MicroImaging) using a 63× NA 1.4 oil plan-Apochromat objective (Carl Zeiss MicroImaging, Inc). All images were collected with a cooled back-thinned CCD camera (MicroMax 512BFT; Princeton Instruments) with a 2× optovar attached using MetaMorph software (Molecular Devices). For population data, 20–40 randomly selected cells were imaged per coverslip. In order to collect velocity data, live cells were imaged twice, 30 seconds apart. Individual cells were imaged at 2- or 5-second intervals for FSM or measurement of edge dynamics, respectively.

### Cell Shape Analysis

Cell morphology was measured by representing cell shapes as polygons, as described [29],[34]. Briefly, cell shapes were extracted manually from phase images using the Magnetic Lasso tool in Photoshop (Adobe) and saved as binary images. Using Celltool, an open source collection of tools for quantifying cell shape [34], polygonal cell outlines were extracted from the binary images and represented as two-dimensional splines, which were then resampled at 200 evenly spaced points to generate the final polygons. To measure cell edge velocities, 200-point polygons were extracted from long movies of individual cells. Displacement vectors between polygons extracted from successive image frames were calculated for each point. The edge velocity at each point was calculated by dividing the component of the displacement vector normal to the cell edge by the time interval at which the images were acquired (5 seconds). The polygon points were numbered for each frame such that the point at the front center of the leading edge was the first point of the polygon (point 0). To measure steady-state cell shapes for a large population of cells, polygons were extracted from a large population of cell images and mutually aligned. Principal modes of shape variation were determined by principal component analysis of the population of polygonal cell outlines, and scaled in terms of the standard deviation of the population for each mode of variation. In addition, cell area, aspect ratio, and left-right asymmetry were measured directly from the aligned polygons. Aspect ratio was measured by dividing the cell width (the cell axis perpendicular to the direction of movement) by the cell length (the cell axis parallel to the direction of movement). Left-right asymmetry was measured by dividing the length of the cell on one side of the cell body by the length of the cell on the other side with the greater length in the denominator, such that the asymmetry measurement was 1 or greater for all cells.

### Measurement of Actin Network Flow

Movement of the actin network was measured using an adaptive multi-frame correlation algorithm as described [26]. Briefly, we used 5-frame averaging (10 seconds) and a correlation template between 11×11 and 21×21 pixels. This method assumes steady-state movement of the actin network within the area of the correlation template over the duration of the temporal window, but keratocytes move too quickly to meet this requirement. Thus, image sequences were converted from the laboratory frame of reference to the cell frame of reference prior to flow tracking [66]. The flow measurements were performed in the cell frame of reference and the resulting flow maps were then transformed back to the laboratory frame of reference. Also, phalloidin speckles were accentuated by applying a spatial band-pass filter to the images before flow tracking.

### Mathematical Modeling

The model consists of coupled sub-models for (i) viscous flow of the F-actin network, (ii) myosin transport, (iii) adhesion density and (iv) F-actin density. We solved the equations of these sub-models (described in detail in Text S1) for different values for the adhesion drag coefficent using the GPL-licensed software FreeFem++ (available for download at http://www.freefem.org) designed to solve partial difference equations using finite element methods. To dynamically compute cell shape, we used the following iteration procedure: at each iteration step, myosin, F-actin, and adhesion densities and centripetal actin flow were simulated until all densities and the flow reached steady state. Then, the boundary mesh was advanced/retracted using the forward Euler method in the locally normal direction with the rate 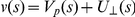, where 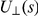 is the normal component of the simulated flow pattern 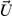 at the cell boundary and 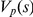 is the rate of actin polymerization. After the shape change, the lamellipodial area was remeshed and the density and flow simulations were repeated until the iterations converged to a stable shape.

## Supporting Information

Figure S1 **Cell-substrate adhesion strength increases with increasing PLL-PEG-RGD concentration.** Keratocytes plated on glass coverslips coated with a range of PLL-PEG-RGD concentrations were centrifuged, upside down, at 1600 × g for 10 minutes. The number of cells attached to each surface was counted before and after centrifugation, and the average percentage of cells that remained attached following centrifugation for three trials is plotted versus PLL-PEG-RGD concentration. Error bars indicate standard error of the mean.
(TIF)

Figure S2 **Variations in cell shape increase at high adhesion strengths.** Front velocity, area, aspect ratio, and left-right asymmetry are plotted over time for individual cells plated on low (top row), intermediate (middle row), and high (bottom row) adhesion strength surfaces (0.8, 4, and 500 μg/ml PLL-PEG-RGD, respectively). N  =  8 cells for each population; each individual cell within the three populations is represented by a distinct line color for all four measurements. The thick red lines indicate the cells shown in Figure 2.
(TIF)

Figure S3 **Simulated myosin and retrograde flow patterns for a generic input cell shape.** Coupled myosin and flow distributions were computed on a fixed, generic cell shape at low (left), medium (center) and high (right) adhesion strengths. (A) Simulated myosin distributions. (B) Simulated actin retrograde flow maps. Color-coded arrows show local flow direction and magnitude (hot colors correspond to faster flow). (C) Distributions of the computed normal component of the centripetal flow around the boundary (blue), polymerization rate (red) and net protrusion/retraction rate (black).
(TIF)

Figure S4 **Simulated adhesion and actin filament distribution patterns for a generic input cell shape.** Coupled adhesions and actin distributions were computed on a fixed, generic cell shape at low (left), medium (center) and high (right) adhesion strengths. (A) Simulated adhesion distributions. (B) Simulated F-actin distributions. (C) Distributions of the computed adhesion (green) and F-actin (red) densities around the cell boundary. Units are non-dimensionalized (n.d).
(TIF)

Figure S5 **Simulated actin network flow patterns for different adhesion strengths and myosin activities.** Nine cell shapes correspond to nine conditions: low, medium, and high adhesion (left, center and right column, respectively) and blebbistatin-treated, control and calyculin-treated cells (lower, center and top row, respectively). Actin retrograde flow was simulated for the nine different cell shapes using the indicated values for the adhesion drag coefficient *ζ* and the myosin force coefficient *k*. See Text S1 for a list of all parameter values. Local flow is indicated by color-coded arrows (hot colors correspond to faster flow).
(TIF)

Figure S6 **The effects of calyculin A on cell speed and area are reduced when myosin contraction is inhibited with blebbistatin.** Histograms that display the distribution of cell speed and area are show for control cells (A) as well as cells treated with 10 μM blebbistatin (B), 10 nM calyculin A (C), or 10 μM blebbistatin + 10 nM calyculin A (D).
(TIF)

Figure S7 **Average measured actin network flow rates at varying adhesion and myosin strengths.** Average actin polymerization rates (red lines) and actin retrograde flow rates (blue lines) measured in populations of cells treated with calyculin A (top row) or blebbistatin (bottom row) are plotted for each point around the cell perimeter. The gray lines are the effective expansion/retraction rates calculated by adding the measured actin polymerization and retrograde flow rates. Error bars indicate standard error of the mean. Measurements from control cells, shown in Figure 6, are shown here for comparison (middle row).
(TIF)

Figure S8 **Simulated actin network flow maps and myosin, adhesion, and actin distributions for the case where the adhesion drag coefficient ζ decreases with increasing adhesion density.** (A) Spatial distribution of the adhesion drag coefficient ζ. (B) Simulated myosin distributions. (C) Simulated actin retrograde flow maps. The direction and magnitude of actin network movement with respect to the underlying substrate is indicated color-coded arrows; hot colors correspond to faster flow. (D) Distributions of the computed normal component of the centripetal flow around the cell boundary (blue), polymerization rate (red), and net expansion/retraction rate (black). The centripetal flow rates at the cell boundary were taken from the simulated flow maps shown in part B. The actin polymerization rates are the rates required to maintain the input cell shape, given the simulated retrograde flow patterns. (E) Simulated adhesion distributions. (F) Simulated actin distributions. (G) Distributions of the computed adhesion (green) and actin (red) densities around the cell perimeter. Units are non-dimensionalized (n.d.). See Text S1 for simulation parameters.
(TIF)

Figure S9 **Simulated actin network flow maps and myosin, adhesion, and actin distributions for the case where the adhesion drag coefficient ζ increases with increasing adhesion density.** (A) Spatial distribution of the adhesion drag coefficient ζ. (B) Simulated myosin distributions. (C) Simulated actin retrograde flow maps. The direction and magnitude of actin network movement with respect to the underlying substrate is indicated color-coded arrows; hot colors correspond to faster flow. (D) Distributions of the computed normal component of the centripetal flow around the cell boundary (blue), polymerization rate (red), and net expansion/retraction rate (black). The centripetal flow rates at the cell boundary were taken from the simulated flow maps shown in part (B). The actin polymerization rates are the rates required to maintain the input cell shape, given the simulated retrograde flow patterns. (E) Simulated adhesion distributions. (F) Simulated actin distributions. (G) Distributions of the computed adhesion (green) and actin (red) densities around the cell perimeter. Units are non-dimensionalized (n.d.). See Text S1 for simulation parameters.
(TIF)

Movie S1 **A keratocyte crawling at intermediate adhesion strength.** The cell is crawling on a glass surface coated with 4 μg/ml PLL-PEG-RGD. The cell is fan-shaped, with clearly defined lead and trailing edges, and moves persistently in one direction. The movie is at 30× real time.
(MOV)

Movie S2 **A keratocyte crawling at low adhesion strength.** The cell is crawling on a glass surface coated with 0.8 μg/ml PLL-PEG-RGD. The cell is round and exhibits noisier protrusion and retraction, compared to the cell in Movie S1. The movie is at 30× real time.
(MOV)

Movie S3 **A keratocyte crawling at high adhesion strength.** The cell is crawling on a glass surface coated with 500 μg/ml PLL-PEG-RGD. The cell exhibits traveling waves of protrusion along the leading edge. The movie is at 30× real time.
(MOV)

Movie S4 **An individual cell transitions between low and intermediate adhesion strength migration behaviors.** The cell is crawling on a micro-patterned surface, where the light region has been stamped with an intermediate concentration of PLL-PEG-RGD and the dark region has been back-filled with a lower concentration. Cell area and aspect ratio increase as the cell crosses from the low adhesion region to the intermediate adhesion region. The movie is at 30× real time.
(MOV)

Movie S5 **An individual cell transitions between intermediate and high adhesion strength migration behaviors.** The cell is crawling on a micro-patterned surface, where the light region has been stamped with a high concentration of PLL-PEG-RGD and the dark region has been back-filled with an intermediate concentration. The cell exhibits protrusion waves immediately after crawling onto the high adhesion region. The movie is at 30× real time.
(MOV)

Movie S6
**Dynamical simulations of cell shape for different adhesion drag coefficents recapitulate experimentally observed differences in cell shape.** Cell shape and actin network flow were simulated using an iteration procedure: myosin densities and centripetal actin flow were simulated until the myosin distribution and the flow pattern reached steady state (see Text S1). Then, the boundary mesh was advanced/retracted in the locally normal direction with the rate 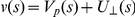, where 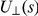 is the normal component of the simulated flow pattern 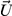 at the cell boundary and 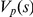 is the rate of actin polymerization. After the shape change, the lamellipodial area was remeshed and the density and flow simulations were repeated until the iterations converged to a stable shape. In the first half of the movie, the adhesion drag coefficient ζ = 0.04 nNs/μm^4^ and the cell converges to a round shape. Halfway through the movie, ζ increases to 0.2 nNs/μm^4^ and the shape evolves to a more elongated shape. The direction and magnitude of local actin network movement with respect to the underlying substrate is indicated by color-coded arrows; hot colors correspond to faster flow.(MOV)

Table S1 **Model variables.**
(PDF)

Table S2 **Constant model parameters.**
(PDF)

Table S3 **Model parameters dependent on adhesion strength.**
(PDF)

Table S4 **Model parameters dependent on myosin strength.**
(PDF)

Table S5 **Model parameters dependent on adhesion and myosin strength.**
(PDF)

Text S1 **Computational model of actin-myosin-adhesion mechanics.**
(PDF)

Text S2 **Supplemental results.**
(PDF)
