# Supplementary figures and images for: An Adhesion-Dependent Switch between Mechanisms That Determine Motile Cell Shape
Source: PLoS Biol. 2011 May 3;9(5):e1001059. doi: 10.1371/journal.pbio.1001059 (PMC3086868; doi:10.1371/journal.pbio.1001059)

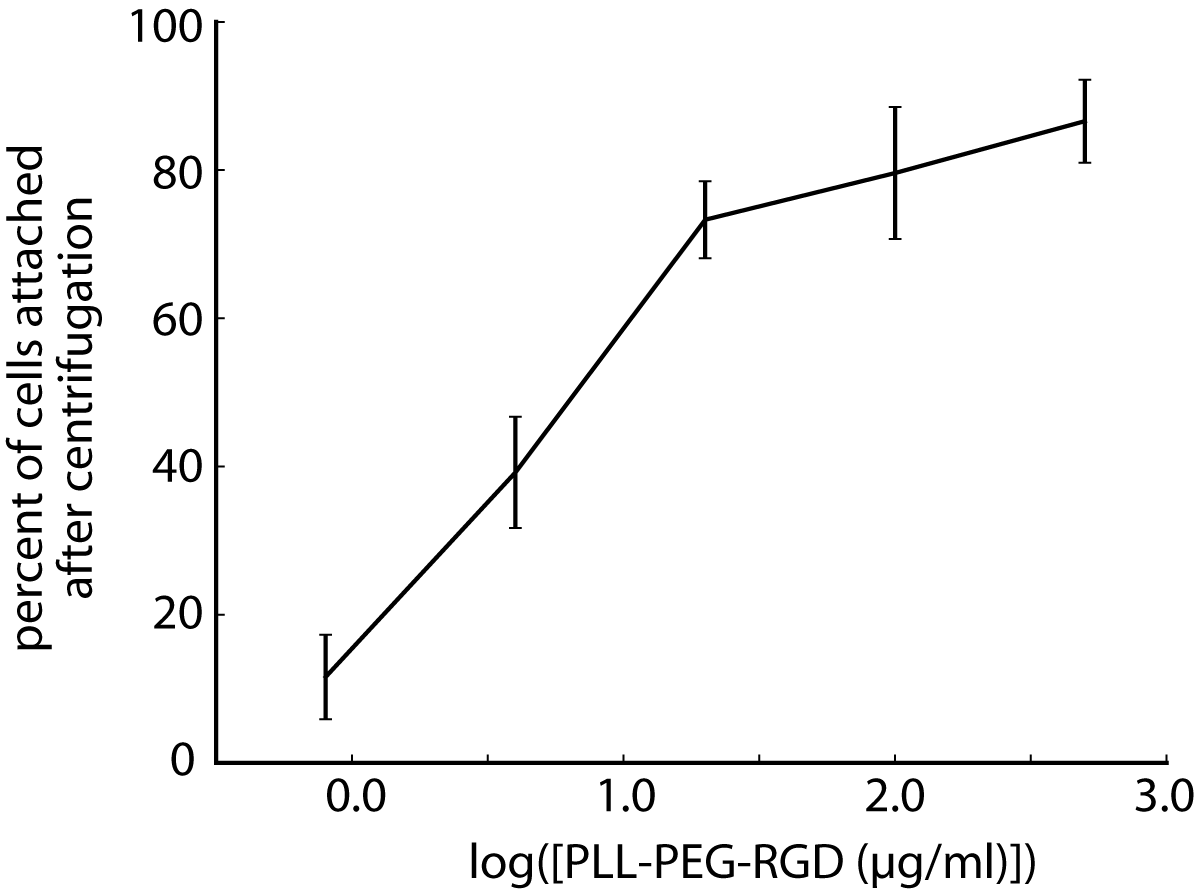

Supplement: Figure S1 — Cell-substrate adhesion strength increases with increasing PLL-PEG-RGD concentration. Keratocytes plated on glass coverslips coated with a range of PLL-PEG-RGD concentrations were centrifuged, upside down, at 1600 × g for 10 minutes. The number of cells attached to each surface was counted before and after centrifugation, and the average percentage of cells that remained attached following centrifugation for three trials is plotted versus PLL-PEG-RGD concentration. Error bars indicate standard error of the mean. (TIF) [file pbio.1001059.s001.tif]

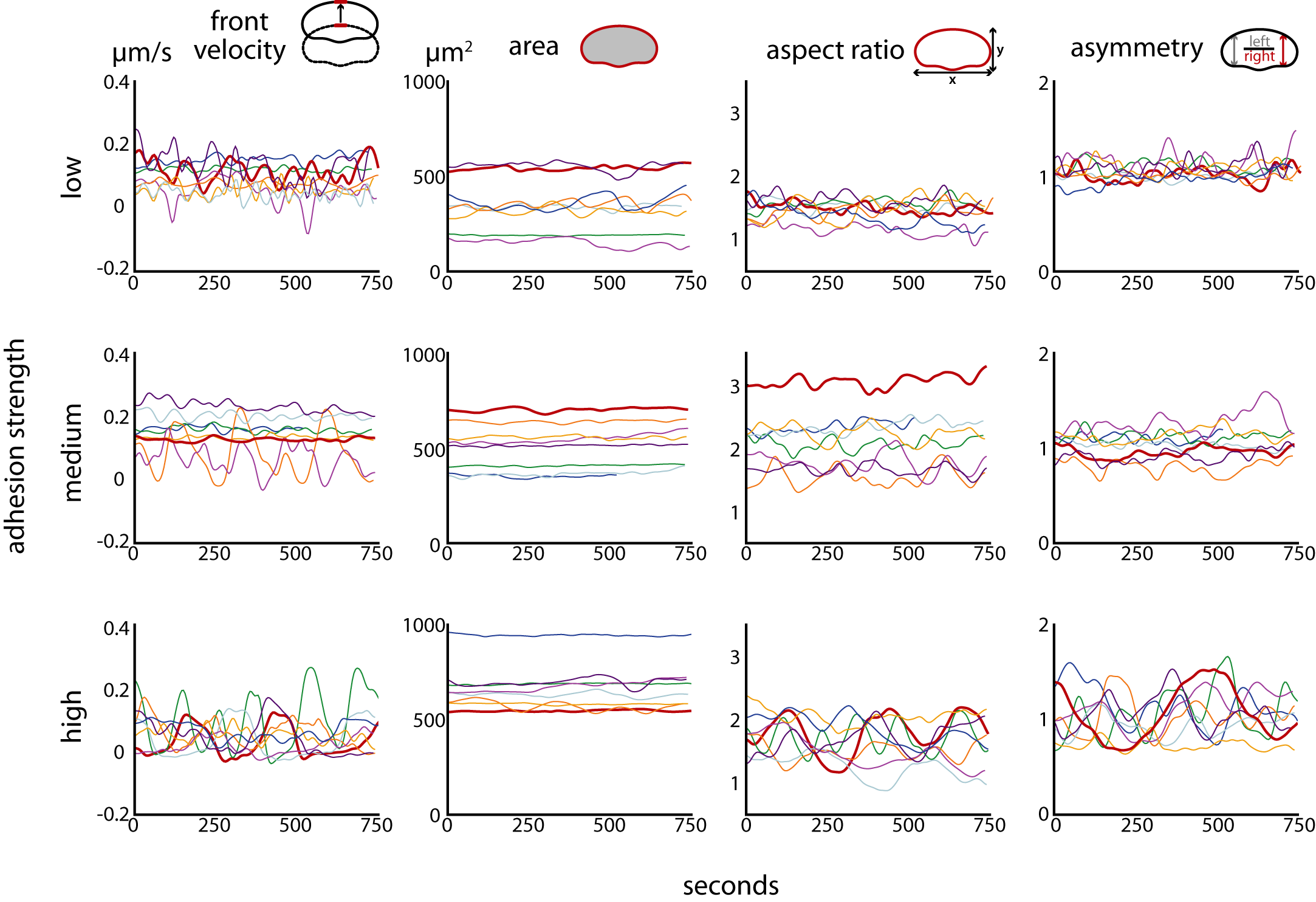

Supplement: Figure S2 — Variations in cell shape increase at high adhesion strengths. Front velocity, area, aspect ratio, and left-right asymmetry are plotted over time for individual cells plated on low (top row), intermediate (middle row), and high (bottom row) adhesion strength surfaces (0.8, 4, and 500 μg/ml PLL-PEG-RGD, respectively). N = 8 cells for each population; each individual cell within the three populations is represented by a distinct line color for all four measurements. The thick red lines indicate the cells shown in Figure 2. (TIF) [file pbio.1001059.s002.tif]

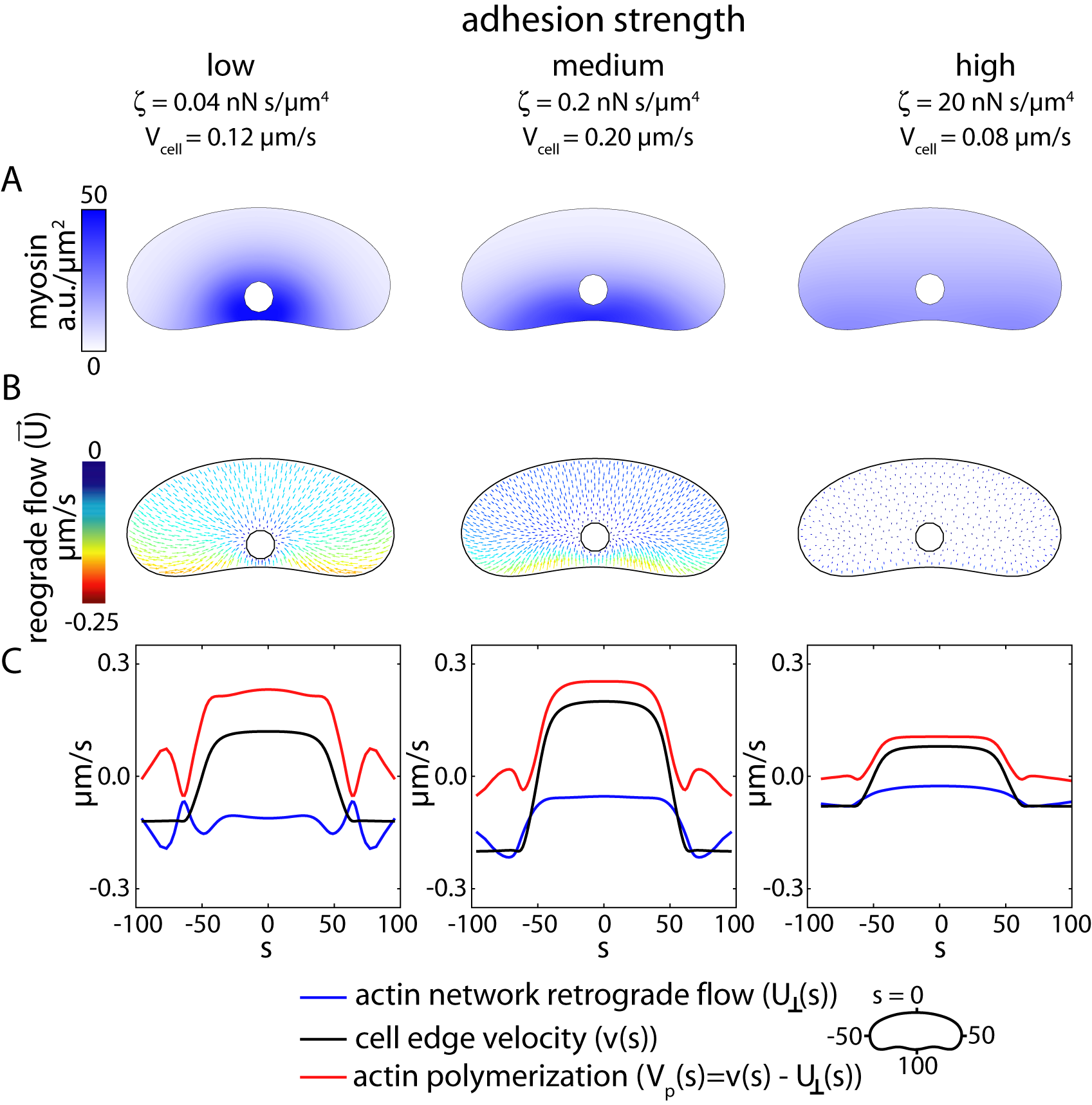

Supplement: Figure S3 — Simulated myosin and retrograde flow patterns for a generic input cell shape. Coupled myosin and flow distributions were computed on a fixed, generic cell shape at low (left), medium (center) and high (right) adhesion strengths. (A) Simulated myosin distributions. (B) Simulated actin retrograde flow maps. Color-coded arrows show local flow direction and magnitude (hot colors correspond to faster flow). (C) Distributions of the computed normal component of the centripetal flow around the boundary (blue), polymerization rate (red) and net protrusion/retraction rate (black). (TIF) [file pbio.1001059.s003.tif]

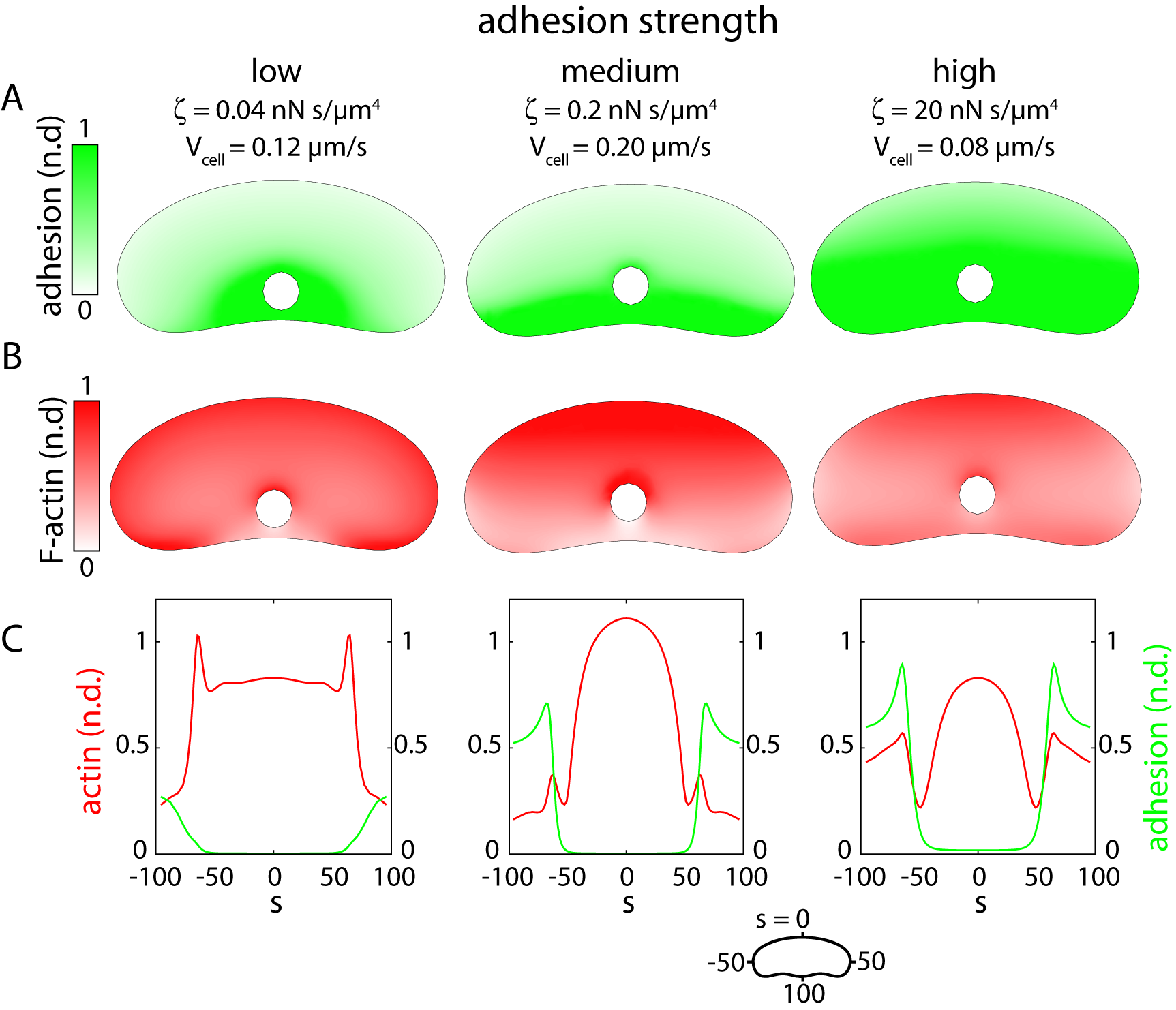

Supplement: Figure S4 — Simulated adhesion and actin filament distribution patterns for a generic input cell shape. Coupled adhesions and actin distributions were computed on a fixed, generic cell shape at low (left), medium (center) and high (right) adhesion strengths. (A) Simulated adhesion distributions. (B) Simulated F-actin distributions. (C) Distributions of the computed adhesion (green) and F-actin (red) densities around the cell boundary. Units are non-dimensionalized (n.d). (TIF) [file pbio.1001059.s004.tif]

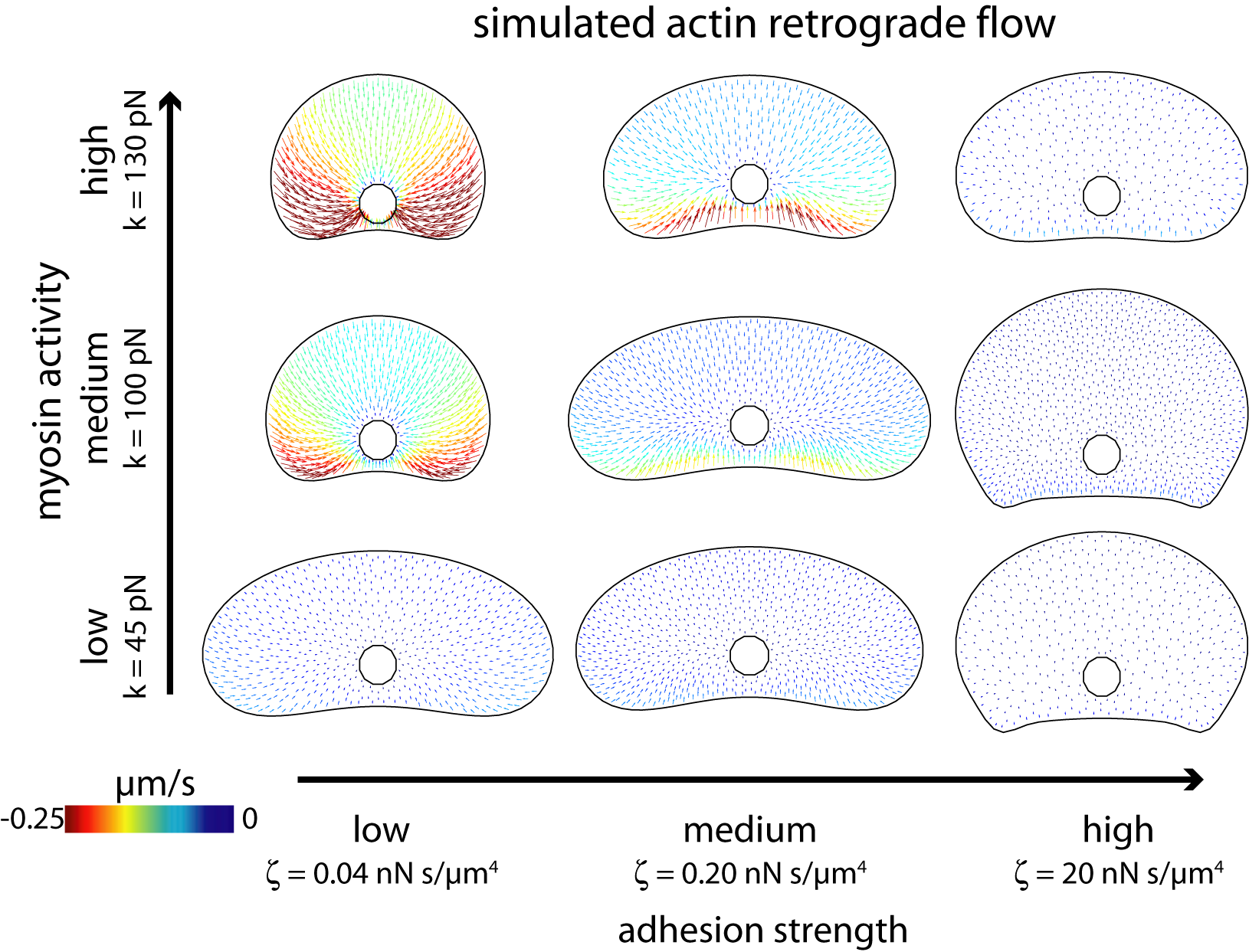

Supplement: Figure S5 — Simulated actin network flow patterns for different adhesion strengths and myosin activities. Nine cell shapes correspond to nine conditions: low, medium, and high adhesion (left, center and right column, respectively) and blebbistatin-treated, control and calyculin-treated cells (lower, center and top row, respectively). Actin retrograde flow was simulated for the nine different cell shapes using the indicated values for the adhesion drag coefficient ζ and the myosin force coefficient k. See Text S1 for a list of all parameter values. Local flow is indicated by color-coded arrows (hot colors correspond to faster flow). (TIF) [file pbio.1001059.s005.tif]

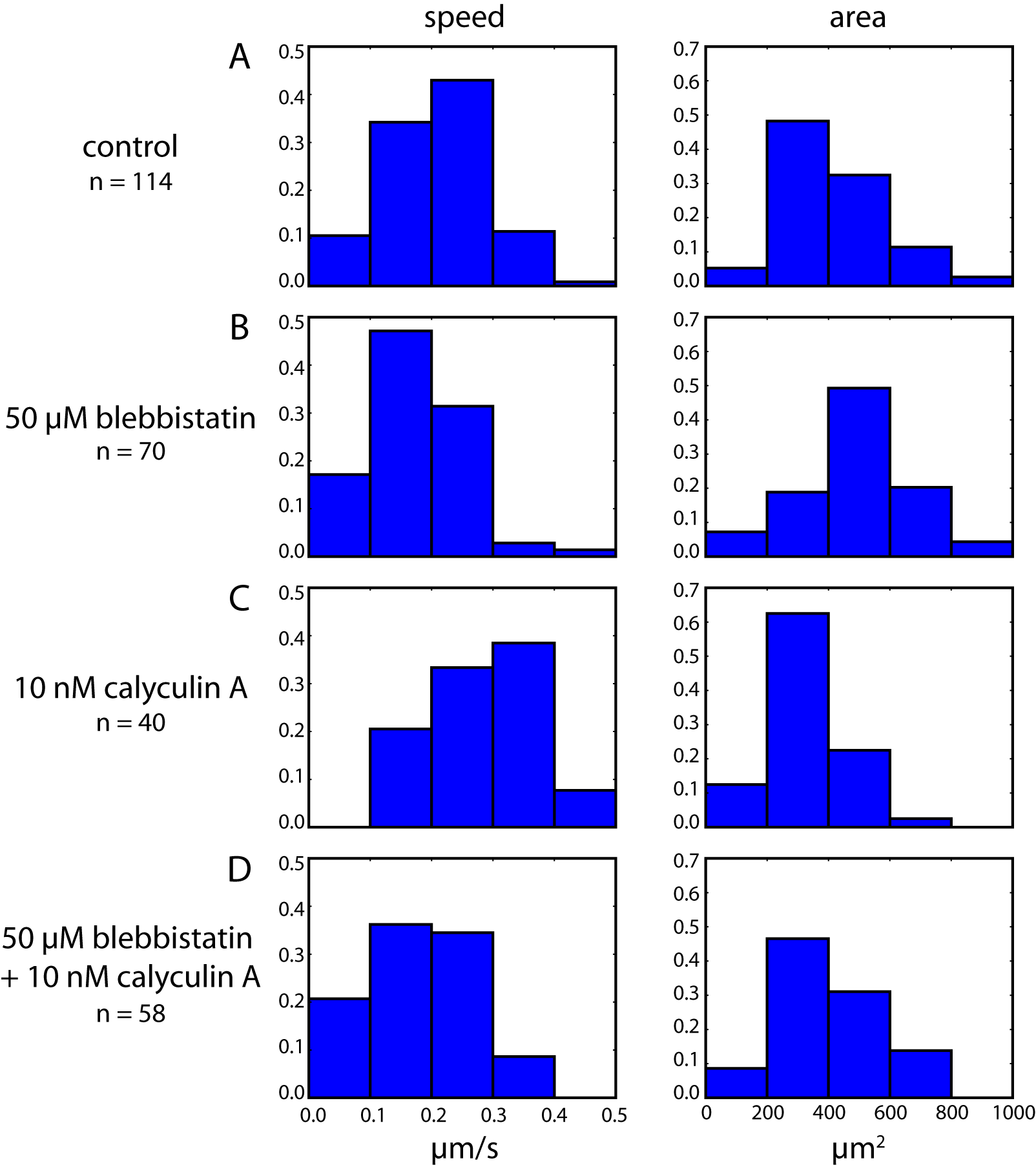

Supplement: Figure S6 — The effects of calyculin A on cell speed and area are reduced when myosin contraction is inhibited with blebbistatin. Histograms that display the distribution of cell speed and area are show for control cells (A) as well as cells treated with 10 μM blebbistatin (B), 10 nM calyculin A (C), or 10 μM blebbistatin + 10 nM calyculin A (D). (TIF) [file pbio.1001059.s006.tif]

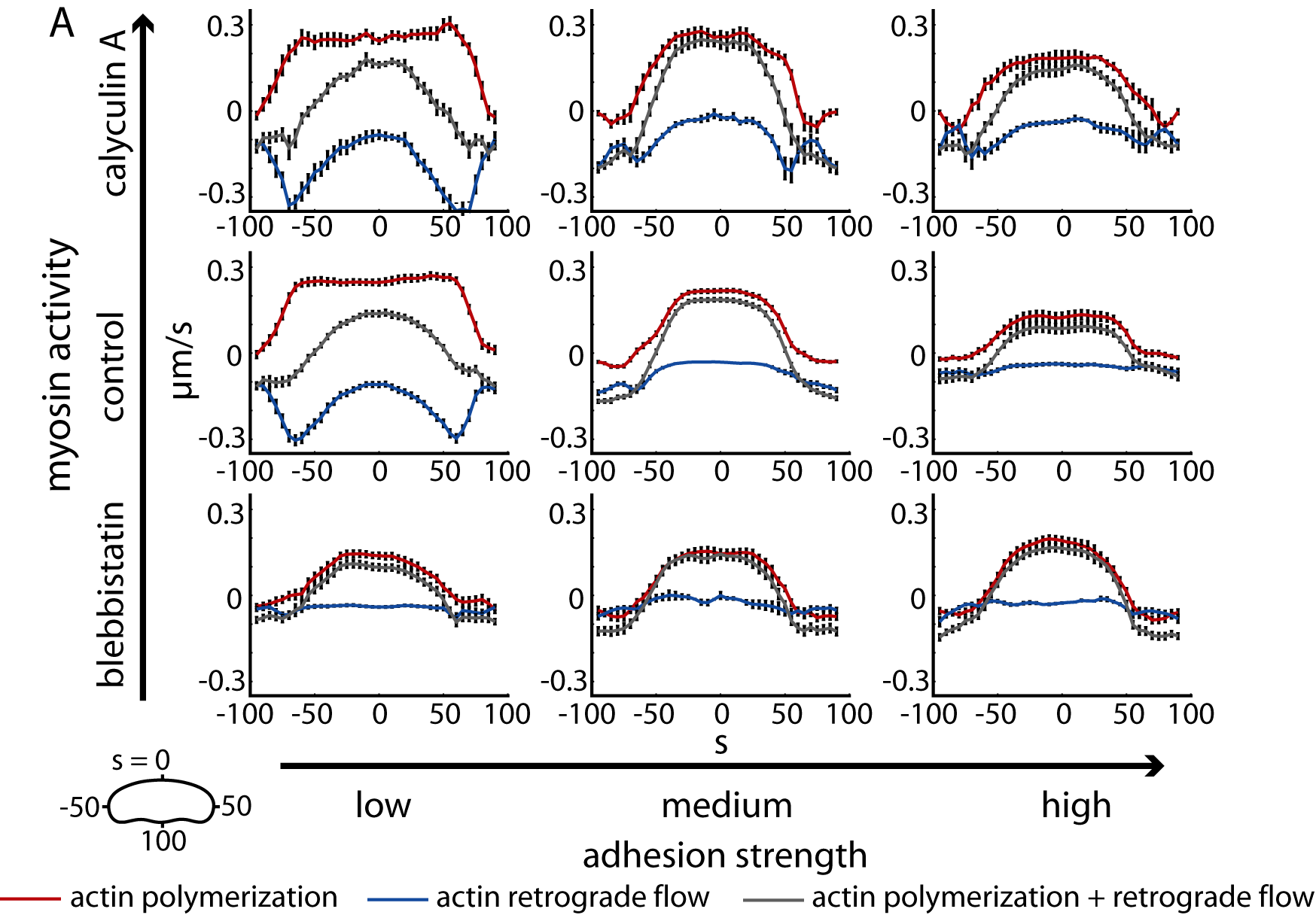

Supplement: Figure S7 — Average measured actin network flow rates at varying adhesion and myosin strengths. Average actin polymerization rates (red lines) and actin retrograde flow rates (blue lines) measured in populations of cells treated with calyculin A (top row) or blebbistatin (bottom row) are plotted for each point around the cell perimeter. The gray lines are the effective expansion/retraction rates calculated by adding the measured actin polymerization and retrograde flow rates. Error bars indicate standard error of the mean. Measurements from control cells, shown in Figure 6, are shown here for comparison (middle row). (TIF) [file pbio.1001059.s007.tif]

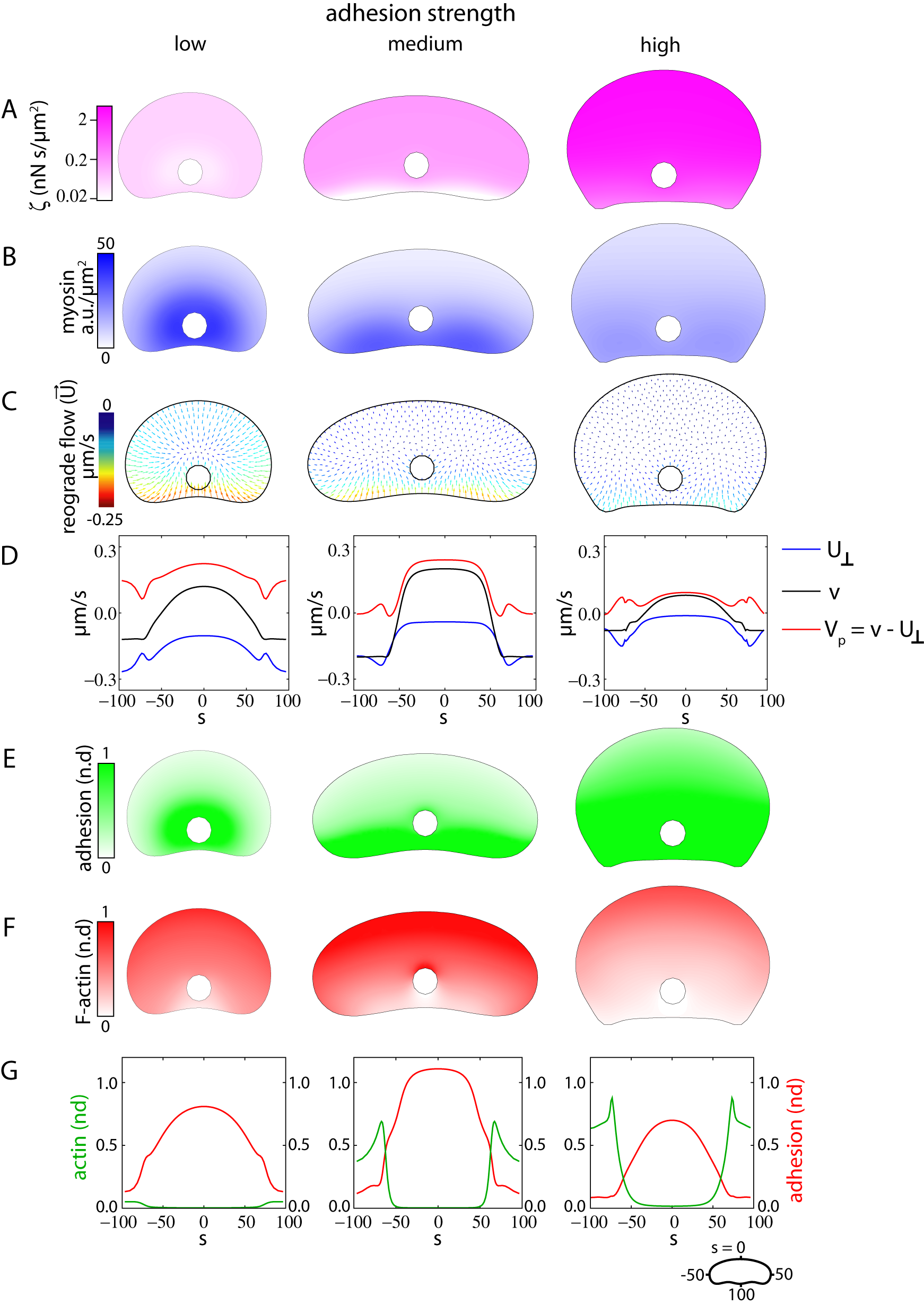

Supplement: Figure S8 — Simulated actin network flow maps and myosin, adhesion, and actin distributions for the case where the adhesion drag coefficient ζ decreases with increasing adhesion density. (A) Spatial distribution of the adhesion drag coefficient ζ. (B) Simulated myosin distributions. (C) Simulated actin retrograde flow maps. The direction and magnitude of actin network movement with respect to the underlying substrate is indicated color-coded arrows; hot colors correspond to faster flow. (D) Distributions of the computed normal component of the centripetal flow around the cell boundary (blue), polymerization rate (red), and net expansion/retraction rate (black). The centripetal flow rates at the cell boundary were taken from the simulated flow maps shown in part B. The actin polymerization rates are the rates required to maintain the input cell shape, given the simulated retrograde flow patterns. (E) Simulated adhesion distributions. (F) Simulated actin distributions. (G) Distributions of the computed adhesion (green) and actin (red) densities around the cell perimeter. Units are non-dimensionalized (n.d.). See Text S1 for simulation parameters. (TIF) [file pbio.1001059.s008.tif]

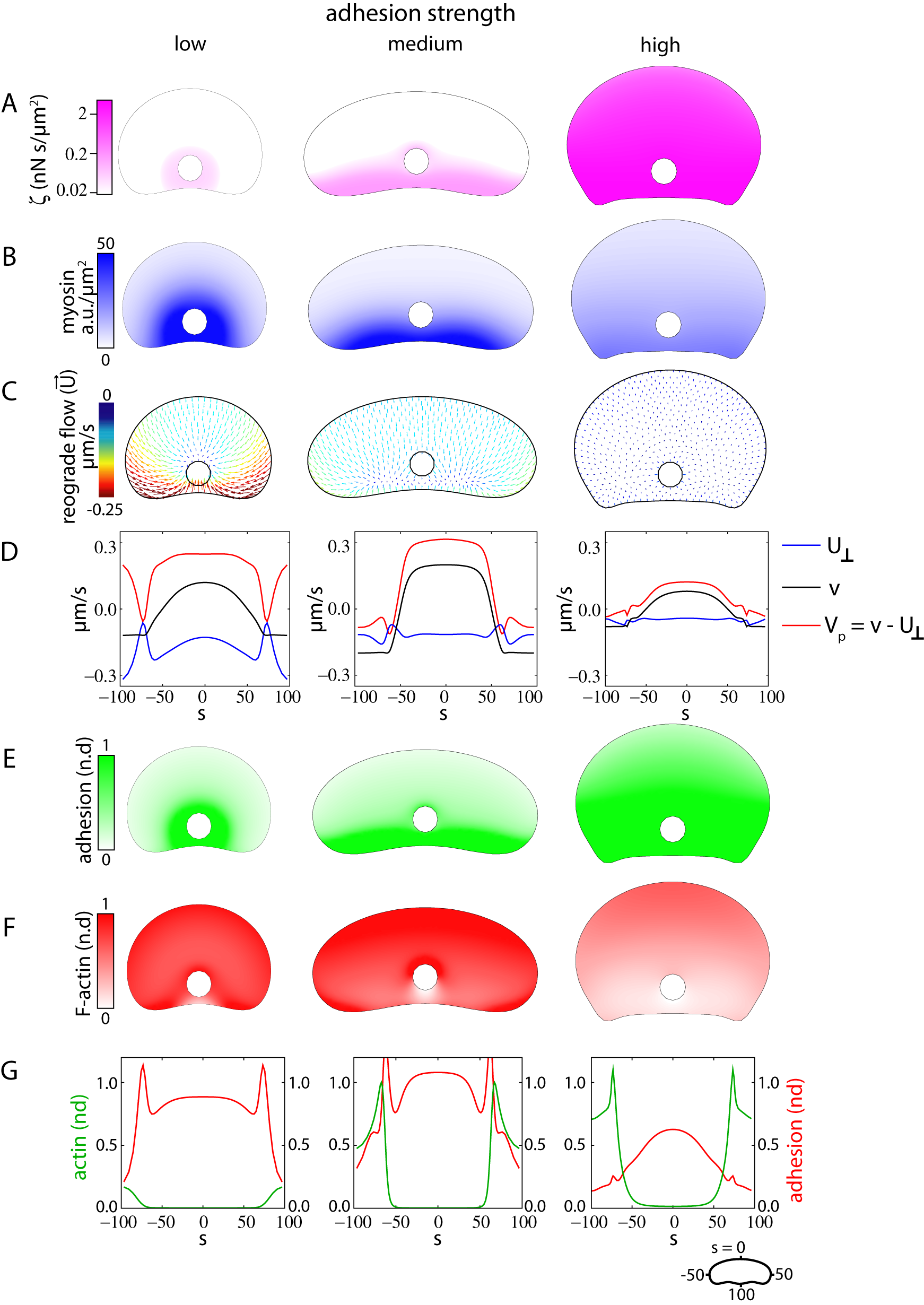

Supplement: Figure S9 — Simulated actin network flow maps and myosin, adhesion, and actin distributions for the case where the adhesion drag coefficient ζ increases with increasing adhesion density. (A) Spatial distribution of the adhesion drag coefficient ζ. (B) Simulated myosin distributions. (C) Simulated actin retrograde flow maps. The direction and magnitude of actin network movement with respect to the underlying substrate is indicated color-coded arrows; hot colors correspond to faster flow. (D) Distributions of the computed normal component of the centripetal flow around the cell boundary (blue), polymerization rate (red), and net expansion/retraction rate (black). The centripetal flow rates at the cell boundary were taken from the simulated flow maps shown in part (B). The actin polymerization rates are the rates required to maintain the input cell shape, given the simulated retrograde flow patterns. (E) Simulated adhesion distributions. (F) Simulated actin distributions. (G) Distributions of the computed adhesion (green) and actin (red) densities around the cell perimeter. Units are non-dimensionalized (n.d.). See Text S1 for simulation parameters. (TIF) [file pbio.1001059.s009.tif]
