# Supplementary material for: An Adhesion-Dependent Switch between Mechanisms That Determine Motile Cell Shape
Source: PLoS Biol. 2011 May 3;9(5):e1001059. doi: 10.1371/journal.pbio.1001059 (PMC3086868; doi:10.1371/journal.pbio.1001059)
Supplement: Table S1 — Model variables. (PDF) [file pbio.1001059.s016.pdf]

Table 1: Model variables.

Variable	Meaning	Dimension
t	time	sec
$\vec{r}$	two-dimensional coordinate	μm
s	arc length along the cell boundary	μm
$M(\vec{r}, t)$	myosin concentration	units/μm^2
$\vec{U}(\vec{r})$	local F-actin flow velocity	μm/s
$\vec{n}(\vec{r})$	local normal unit vector to the lamellipodial edge	non-dimensional
$A(\vec{r}, t)$	adhesion concentration	non-dimensional
$a(\vec{r}, t)$	F-actin density	non-dimensional
$v(s)$	net local protrusion/retraction rate	μm/s
$V_p(s)$	local polymerization rate	μm/s
