# Supplementary material for: An Adhesion-Dependent Switch between Mechanisms That Determine Motile Cell Shape
Source: PLoS Biol. 2011 May 3;9(5):e1001059. doi: 10.1371/journal.pbio.1001059 (PMC3086868; doi:10.1371/journal.pbio.1001059)
Supplement: Table S2 — Constant model parameters. (PDF) [file pbio.1001059.s017.pdf]

Table 2: Constant model parameters.

Parameter	Meaning	Value
$\bar{M}$	total myosin	55 units
D_M	effective myosin diffusion coefficient	0.8 μm^2/s
D_A	small adhesion diffusion coefficient	0.2 μm^2/s
D_a	small actin diffusion coefficient	0.2 μm^2/s
μ	shear F-actin viscosity	2 kPa$\times$s
μ_b	bulk F-actin viscosity	100 kPa$\times$s
S_A	adhesion assembly rate	1 unit/50 s
