# Supplementary material for: An Adhesion-Dependent Switch between Mechanisms That Determine Motile Cell Shape
Source: PLoS Biol. 2011 May 3;9(5):e1001059. doi: 10.1371/journal.pbio.1001059 (PMC3086868; doi:10.1371/journal.pbio.1001059)
Supplement: Table S3 — Model parameters dependent on adhesion strength. (PDF) [file pbio.1001059.s018.pdf]

Table 3: Model parameters dependent on adhesion strength.

Parameter	Meaning	Low	Medium	High
ζ [nN×s/μm^4]	adhesion drag coefficient	0.04	0.2	20
ϑ[non-dim]	flow-coupling coefficient	0.9	0.4	0.2
γ [1/s]	adhesion disassembly rate	1/50	1/150	1/250
υ [1/s]	actin disassembly rate	1/50	1/50	1/100
α [1 unit×μm/s	F-actin assembly rate	2	0.4	0.4
M_0 [non-dim]	threshold myosin density for myosin-mediated actin depolymerization	140	20	200
A_0 [non-dim]	threshold adhesion density for adhesion-dependent inhibition of actin polymerization	0.01	1	0.2
