# Supplementary material for: An Adhesion-Dependent Switch between Mechanisms That Determine Motile Cell Shape
Source: PLoS Biol. 2011 May 3;9(5):e1001059. doi: 10.1371/journal.pbio.1001059 (PMC3086868; doi:10.1371/journal.pbio.1001059)
Supplement: Table S4 — Model parameters dependent on myosin strength. (PDF) [file pbio.1001059.s019.pdf]

Table 4: Model parameters dependent on myosin strength.

Parameter	Meaning	Blebbistatin	Control	Calyculin
k [pN]	myosin force coefficient	45	100	130
