# Supplementary material for: An Adhesion-Dependent Switch between Mechanisms That Determine Motile Cell Shape
Source: PLoS Biol. 2011 May 3;9(5):e1001059. doi: 10.1371/journal.pbio.1001059 (PMC3086868; doi:10.1371/journal.pbio.1001059)
Supplement: Table S5 — Model parameters dependent on adhesion and myosin strength. (PDF) [file pbio.1001059.s020.pdf]

Table 5: Model parameters dependent on adhesion and myosin strength.

Param.	Meaning	Cont Low	Cont Med	Cont High	Caly Low	Caly Med	Caly High	Bleb Low	Bleb Med	Bleb High
$\bar{A}$ [μm^2]	cell area	300	460	513	297	383	397	470	434	456
V_{cell} [μm/s]	cell speed	0.12	0.20	0.08	0.16	0.22	0.08	0.10	0.15	0.16
