# Supplementary material for: An Adhesion-Dependent Switch between Mechanisms That Determine Motile Cell Shape
Source: PLoS Biol. 2011 May 3;9(5):e1001059. doi: 10.1371/journal.pbio.1001059 (PMC3086868; doi:10.1371/journal.pbio.1001059)
Supplement: Text S1 — Computational model of actin-myosin-adhesion mechanics. (PDF) [file pbio.1001059.s021.pdf]

Text S1

Computational model of actin-myosin-adhesion mechanics

Myosin-powered retrograde actin network flow

Experimental and theoretical studies have established that myosin contracts actin arrays and generates contractile stress and that this stress grows with increasing myosin concentration [1, 2]. For simplicity in our model, we neglect the possibility that the myosin stress could be anisotropic, consider it a scalar and make the simplest assumption that the stress, kM, is linearly proportional to the myosin density. Here M is the myosin density and k is the proportionality coefficient (typical force per myosin unit) that depends on blebbistatin/calyculin treatment. The contractile force applied to the actin network is the divergence of the stress, in this case, its gradient, $k\nabla M$. Following [3] we assume that adhesion complexes generate viscous resistance to the flow of F-actin (with velocity $\vec{U}$ in the lab coordinate system). The respective resistance force, $\zeta\vec{U}$, where ζ is the effective drag coefficient that we call adhesion strength, is balanced by the active contractile stress: $\zeta\vec{U} = k\nabla M$.

The simple equation $\zeta\vec{U} = k\nabla M$ does not take into account passive stresses in the F-actin network due to its deformation during the flow. To add these passive stresses, we follow [3] and assume that these stresses have viscous character on the relevant time scale of tens of seconds. The small elastic component of the stress in the lamellipodium can be neglected [3], so we model a combination of the shear and deformation stresses in the F-actin with the formula $\left(\mu_b - \frac{2}{3}\mu\right)(\nabla \cdot \vec{U})I + \mu(\nabla\vec{U} + (\nabla\vec{U})^T)$, where μ and μ_b are the shear and bulk viscosities, respectively, and I is the identity tensor. Adding the divergence of these passive stresses to the myosin and adhesion forces results in the force balance equation determining the flow rate:

$$\left[\left(\frac{1}{3}\mu + \mu_b\right)\nabla\nabla \cdot \vec{U} + \mu\nabla^2\vec{U}\right] + k\nabla M = \zeta\vec{U} \qquad (1)$$

The boundary condition is the zero pressure at the free lamellipodial boundary:

$$\vec{n}\left[\left(\mu_b - \frac{2}{3}\mu\right)(\nabla \cdot \vec{U})I + \mu(\nabla\vec{U} + (\nabla\vec{U})^T) + kM\right] = 0 \qquad (2)$$

Here $\vec{n}$ is local normal unit vector to the lamellipodial boundary. This model assumes that the F-actin

viscosity is spatially constant, independent of the F-actin density, which simplifies the analysis. A more detailed assumption of the viscosity being a function of the F-actin density does not change the qualitative pattern of the actin flow [3].

We also assume that there is a small pressure δ at the cell body boundary (shown as the circular domain inside the lamellipodium):

$$\vec{n}\left[\left(\mu_b - \frac{2}{3}\mu\right)(\nabla \cdot \vec{U})I + \mu(\nabla\vec{U} + (\nabla\vec{U})^T) + kM\right] = \delta \tag{3}$$

The rationale for such pressure is that the cell body is squeezed at the top by the membrane tension, so the cell body tends to expand to the sides and compress the underlying actin gel. This pressure is not critical: without it (if $\delta = 0$) all results are valid, except the predicted actin flow at the center of the rear becomes a bit too fast. There are no constraints on the flow velocity at the cell body boundary.

Adhesion strength

Cell-substrate adhesion is thought to be mediated by a mechanism in which adhesion molecules act as "molecular clutches" that couple the actin network to the underlying substrate [4, 5]. The strength of cell-substrate adhesion, which in our model is described by the adhesion drag coefficient ζ, depends on the number of clutches bound to both the actin network and the substrate. Cell-substrate adhesion is mediated by integrin binding of extracellular matrix proteins, and so ζ depends on the density of integrin molecules and the integrin binding affinity for both the ECM on the outside of the cell and for additional adhesion proteins that link integrins to the actin network on the inside of the cell. The spatial distribution of integrin density and affinity states has not been well-characterized in keratocytes, and so as a first pass, we make the simple assumption that ζ is spatially constant (Figures 5, 9, and 11 in the main text). However, the spatial distribution of mature adhesions, as marked by the adhesion protein vinculin, is heterogeneous (see Figure 10 in the main text). The relationship between the size and age of adhesions and adhesion strength has not been well characterized. The simplest possibility is that increased density of adhesion molecules correlates with a higher density of molecular bonds and protein friction. Alternatively, increased density of integrin $\alpha_V\beta_3$ in older adhesions has been shown to be correlated with increased turnover of integrin $\alpha_V\beta_3$ in the cell rear in migrating mouse melanoma cells [6] and estimates of adhesion strength in migrating keratocytes suggest that adhesion strength is greater in the front of the cell [7], despite increased densities of adhesion proteins in the cell rear [8, 9]. Therefore, we have considered both the case where the magnitude of ζ decreases with increasing adhesion density and the case where ζ increases

with increasing adhesion density (Supplemental Figures 8 and 9, respectively). In the case where adhesion strength decreases with adhesion density, the adhesion friction coefficient $\zeta = \zeta_{small} + \frac{2\zeta_0}{1+(A/A_\zeta)^2}$, where ζ_{small} is the minimal friction coefficient, ζ_0 equals to the value of ζ for the homogeneous model, A_ζ sets the threshold adhesion density at which ζ is strongly affected by the adhesion density, and A is the adhesion density (see the model for adhesion dynamics below). For the case where adhesion strength increases with adhesion density, $\zeta = \zeta_{small} + \frac{2\zeta_0(A/A_\zeta)^2}{1+(A/A_\zeta)^2}$.

Myosin transport

Following [3], we assume that myosin molecules associate and move with the F-actin network. Myosin molecules detach from the F-actin, diffuse in the cytoplasm and reattach. Here, we assume that detachment and reattachment is rapid, in which case the system of equations for the actin-associated and diffusing myosin molecules [3] reduces to just one equation for the actin-associated myosin, in which the rapid cycles of the detachment, diffusion in the cytoplasm and reattachment effectively result in slow diffusion of the actin-associated myosin added to the myosin movement due to coupling with the F-actin:

$$\frac{\partial M}{\partial t} = D_M \nabla^2 M - \nabla \cdot ((\vec{U} - \vec{V_{cell}})M) \tag{4}$$

Here D_M is the effective diffusion coefficient, and $\vec{V}_{cell}$ is the velocity of the cell migration. Note that we calculate the myosin distribution in the framework of the moving cell. In the cell frame of reference, the F-actin moves backward with speed V_{cell} as well as flowing centripetally with velocity $\vec{U}$. Myosin moves with the local actin network velocity, so the vector sum of V_{cell} and $\vec{U}$ gives the net drift rate for myosin. The boundary condition for the myosin transport is the zero flux of myosin through the lamellipodial boundary:

$$\vec{n} \cdot \left[D_M \nabla M - M(\vec{U} - \vec{V}_{cell}) \right] = 0 \tag{5}$$

This formulation is valid if the the majority of myosin molecules are attached to the actin network and move with the actin network, as previously demonstrated for keratocytes (Svitkina et al., 1997). The effective diffusion rate and explicit processes of myosin turnover are not crucial for the model results.

Adhesion dynamics

We assume that adhesion molecular complexes (described by the density A) appear across the lamellipodium with the constant rate S_A and disassemble with the constant rate γ. Furthermore, following [10], we assume that there is slippage between molecules of the adhesion complexes, so the adhesion complexes are dragged on the surface by the F-actin flow with the velocity $\vartheta\vec{U}$ which is collinear with the F-actin flow but is slower ($0 < \vartheta < 1$ is the flow-coupling coefficient). For numerical stability, to smoothen the adhesion distribution, we add a slow diffusion of the adhesion complexes with the small diffusion coefficient D_A. Besides the computational advantage, the diffusion term reflects small stochastic displacements of the adhesion molecules. Thus, the adhesion density in the cell frame is governed by the equation:

$$\frac{\partial A}{\partial t} = D_A \nabla^2 A - \nabla \cdot ((\vartheta \vec{U} - \vec{V}_{cell})A) + S_A - \gamma A \tag{6}$$

A kinematic argument similar to that for the myosin sub-model explains the drift rate $(\vartheta\vec{U} - \vec{V}_{cell})$ of the adhesions, and also similarly to the myosin case, the boundary condition is the zero flux:

$$\vec{n} \cdot \left[D_A \nabla A - A(\vartheta \vec{U} - \vec{V}_{cell}) \right] = 0 \tag{7}$$

Note that this sub-model computes the adhesion *density* distribution rather than the distribution of mechanical adhesion *strength*. The relationship between adhesion density distribution and mechanical adhesion strength is described above.

F-actin dynamics

The F-actin (density a) drifts with velocity $(\vec{U} - \vec{V}_{cell})$ in the moving cell framework and disassembles across the lamellipodium with the rate v. Myosin contraction has been shown to accelerate the disassembly of the F-actin network [11], so we assume that v increases with increasing myosin contraction. For numerical stability, to smoothen the actin distribution, we add a slow diffusion characterized by the small diffusion coefficient D_a to the actin drift. Besides the computational advantage, the diffusion term reflects small stochastic fluctuations of the actin filaments. The F-actin density in the cell frame is described by the equation:

$$\frac{\partial a}{\partial t} = D_a \nabla^2 a - \nabla \cdot \left(\left(\vec{U} - \vec{V}_{cell} \right) a \right) - va \left(1 + (M/M_0)\right) \tag{8}$$

We assume that actin filaments assemble at the lamellipodial edge with a rate affected by antagonism between adhesions and the branched actin network [12, 13]. Specifically, we use the formula:

$$\vec{n} \cdot \left[D_a \nabla a - a \left(\vec{U} - \vec{V}_{cell} \right) \right] = \frac{\alpha}{1 + (A/A_0)} \tag{9}$$

Here, the right side of the equation describes the influx of F-actin at the boundary, α, which is inhibited by the adhesion density (factor in the denominator).

Model parameters

The model variables and parameters are listed in Tables I-V. For the actin flow sub-model, we use the parameter values estimated for the keratocyte lamellipodium in (Rubinstein et al. 2009). We use a typical value for the shear viscosity, $\mu = 2$ kPa×s. The bulk viscosity is normally higher than the shear viscosity, as the gels are more resistant to compression than shear. We use the value $\mu_b = 100$ kPa×s. In order for the myosin stress to generate the observed flow of the order of ∼0.1 μm/s, the stress has to be ∼100 pN/μm^2, which is also realistic considering known estimates of the traction force and number of myosin molecules. Thus, we use the force $k = 100$ pN. In the calculations, we multiply the viscosities and parameter k by the characteristic thickness of the lamellipodium $h = 0.2$ μm in order to convert the 3D stress derivatives into the 2D surface force densities. For the adhesion drag coefficient, we use $\zeta = 0.2$ nN×s/μm^4, of the same order of magnitude as the estimate in [3]. To non-dimensionalize the equations, we choose the characteristic lamellipodial size, ∼10 μm, as the scale of distance and the cell speed, ∼0.2 μm/s as the scale of the velocity, so 10 μm/0.2 μm/s ∼50 s is the scale of time. We choose $\mu h \vec{V}_{cell}$ ∼100 pN as the scale of force.

We use the total amount of myosin $\bar{M} = 55$ of non-dimensional units, so that the average myosin density (total myosin divided by the cell area) multiplied by the characteristic myosin force per square micron gives typical known contractile stress. Note that the total amount of myosin is conserved in the model: when the cell area goes up/down compared to the control, the average myosin density decreases/increases. We used the diffusion terms $D_M = 0.8$ μm^2/s, $D_A = 0.2$ μm^2/s and $D_a = 0.2$ μm^2/s which guaranteed that the diffusion terms were significantly smaller than the drift terms.

The observations indicate that many individual adhesion complexes disappear within the lamellipodium, but many others survive from the front to the rear, so we chose the adhesion disassembly rate $\gamma = 1/150$ s, comparable but higher than the inverse characteristic time for the cell migration. In order to maintain the non-dimensionalized adhesion density of the order of 1, we use the adhesion assembly rate $S_A = 1$ unit/50 s. We chose the flow-coupling coefficient $\varphi = 0.4$ assuming that the adhesion molecules on the average slip to comparable degrees relative to the surface and actin network.

Multiple observations suggest that the F-actin disassembles within tens of seconds in the lamellipodium [11], so we choose the actin disassembly rate $v = 1/50$ s. In order to maintain the non-dimensionalized actin density of the order of 1, we use the actin assembly rate $\alpha = 0.4$ units$\times\mu$m/s. Numerical experiments showed that a realistic spatial F-actin distribution evolves if the threshold myosin amount that significantly accelerates actin disassembly is $M_0 = 20$, and the characteristic adhesion density affecting actin assembly is $A_0 = 1$.

The parameters above are for the control cells. For low/high adhesion, the parameter ζ was decreased/increased as shown in Table III. Similarly, when myosin was inhibited/enhanced, the parameter k was lowered/elevated as shown in Table IV. We changed the flow-coupling coefficient to 0.9 and 0.2 for low/high adhesion conditions, respectively, assuming that as adhesion strength increases, adhesion molecules associated more tightly with the surface and slip more relative to the actin network. The adhesion disassembly rate was increased to 1/50 s and decreased to 1/250 s for low/high adhesion conditions, respectively. The actin disassembly rate was decreased to 1/100 s for high adhesion conditions; otherwise, the actin density decreases too much in the cell interior due to the very slow centripetal flow. Perhaps higher adhesion contributes to enhanced stability of existing actin filaments. Finally, multiple numerical experiments demonstrated that realistic actin distributions are achieved if myosin affects actin depolymerization less at both low and high adhesion compared to the control case (Table III).

In the simulations, we used various cell areas and speeds given by the experimental measurements for the nine conditions (Table V).

Simulation of actin-myosin-adhesion dynamics and predicting respective densities

We generated numerically fixed cell shapes similar to those observed experimentally, and used the LGPL-licensed software FreeFem++ (designed to solve Partial Differential Equations using finite element methods; available for download at www.freefem.org) to solve Eqs. 1-8 as follows. First, Eqs. 1-5 for flow velocities

and myosin distributions were solved numerically with initial conditions of uniformly spread myosin and zero flow, resulting, after transients decreased almost to zero, in a stable myosin distribution $M(\vec{r})$ and flow field $\vec{U}(\vec{r})$. Then, using the resulting steady flow field, we solved Eqs. 6-7 to find the stable adhesion distribution $A(\vec{r})$. Finally, using the resulting steady flow field and myosin and adhesion distributions, we solved Eqs. 8-9 to find the stable F-actin distribution $a(\vec{r})$.

Prediction of the polymerization rate and net protrusion/retraction rate

We assume that the cell boundary is determined by the Graded Radial Extension model [14]: the rate of the net protrusion and retraction of the boundary is graded, and in the steady state, the cell shape is determined by the angle between the local normal vector to the cell boundary and the direction of the cell migration $\theta(s)$, which is related to the protrusion/retraction rate, $v(s)$, by the formula:

$$\cos\left(\theta\left(s\right)\right) = v\left(s\right)/v\left(0\right) \tag{10}$$

Here s is the arc length along the cell boundary; we calibrate it so that $s = 0$ corresponds to the center front, $s = \pm 50$ corresponds to the sides, and $s = \pm 100$ corresponds to the center rear. An additional constrain is that $v(0) = V_{cell}$. Thus, we calculate the graded protrusion/retraction rate for a given cell shape using the formula:

$$v\left(s\right) = V_{cell}\cos\left(\theta\left(s\right)\right) \tag{11}$$

Then, from the computed centripetal flow $\vec{U}$ at the boundary, we find the projection of the flow onto the inward normal to the cell boundary, $U_{\perp} = -\vec{U}\cdot\vec{n}$, and note that the protrusion/retraction rate, $v(s) = V_p(s) + U_{\perp}(s)$, where v_p is the actin polymerization rate. Thus,

$$V_p\left(s\right) = v\left(s\right) - U_{\perp}\left(s\right) \tag{12}$$

We use Eqs. 10-11 to calculate the net protrusion/retraction rate and actin polymerization rates for given cell shapes.

Dynamic cell shape

According to [13], the actin polymerization rate, according to model, is given by the formula: $V_p(s) = V_0 \times (1 - \frac{T}{f_{stall} D(s)})^w)$, where V_0 is the free polymerization rate proportional to the G-actin density, f_{stall} is the stall force per filament, T is the membrane tension,$w >> 1$ is the parameter characterizing the force-velocity relation for actin filaments, and $D(s)$ is the local density of the pushing barbed ends proportional to the F-actin density at the boundary. Because of the strong inequality $w >> 1$ (previously published experiments suggest that $w \sim 8$ [13]), the polymerization rate is predicted to be a step-function-like along the leading edge: almost constant along the front and dropping abruptly to very small values at the sides and rear. This prediction is in agreement with both our calculations (Figure 5) and experimental data (Figure 7). The membrane tension T, which at the moment we cannot measure directly, is the parameter regulating where along the boundary the polymerization rate drops.

In addition, the membrane tension, lamellipodial area and adhesion strength are interdependent: according to the observations, the area increases with the adhesion strength. We assume that the reason is the more significant adhesion free energy decrease when the lamellipodium spreads on stronger adhesion surfaces [15]. At the same time, greater lamellipodium spreading likely comes at the expense of pulling the plasma membrane off the cell body (causing squeezing of the cell body) and dynamic shift of the equilibrium between the intracellular and plasma membrane toward the greater plasma membrane area, which causes an increase of the membrane tension [16].

Thus, to simulate the cell shape dynamically, we assume: (i) There is a constant target cell area at given adhesion strength (the area is an increasing function of the adhesion strength). (ii) When area increases, the membrane tension increases, and the protrusion decreases. Specifically, we approximate the polymerization rate along the boundary with the function:

$$V_p(s) = V_0 \times g(s) + p(A) \tag{13}$$

Here $g(s)$ is a smoothened step-function: it is equal to 1 at the leading edge up to the middle of the sides, and equal to zero at the rear. The term $p(A)$ is constant along the boundary and represents a small additional polymerization at the rear and front. However, this constant depends on the cell area: it decreases when the cell area increases. Also, this constant increases with the adhesion strength which leads to the increase of the equilibrium cell area with the adhesion strength. The function $p(A)$ is calibrated so that the target area of the cell stays constant in the dynamic simulations. In the future, we will upgrade the model by introducing a more detailed dependence of the polymerization rate on the membrane tension

and area.

Using these assumptions, the dynamic iterative procedure of the cell shape simulation is as follows: 1) We solved coupled Eqs. 1-4 on a given cell shape until the steady stable myosin and flow distributions evolved. 2) From the steady centripetal flow distribution $\vec{U}$ at the boundary, we computed the projection of the flow onto the inward normal to the cell boundary, $U_\perp$, and the protrusion/retraction rate, $v(s) = V_p(s) + U_\perp(s)$, where V_p is given by Eq. 12. Parameter $p(A)$ was adjusted to keep the cell area constant. 3) We evolved the cell boundary using the computed rate $v(s)$ (by using forward Euler method to displace nodes of the discrete grid on the boundary). 4) We re-meshed the boundary and the 2D domain encircled by it and repeated the steps 1-3. Repeating this iterative procedure led to convergence of the cell shape to a stable one.

Model Results

From dimensional and linear stability analysis of the coupled actin-myosin-adhesion dynamics, we conclude that the order of magnitude of the centripetal flow of actin turns out to be $\sim \frac{kM}{\sqrt{\zeta\mu_b}}$. Thus, the flow is proportional to the myosin strength, and is a decreasing function of the adhesion strength. This prediction is illustrated by Supplemental Figure 3: on the fixed cell shape, the overall magnitude of the flow decreases when adhesion strength increases. Similarly, Supplemental Figure 5 shows that the flow accelerates/decelerates when the myosin strength goes up/down.

In the case where ζ is spatially constant, the resulting actin network flow patterns (the flow is slow at the front and sides, and fast at the rear) compare very well with the experimental data (compare Figures 5 and 7). If adhesion strength is high and/or myosin activity is low, then the centripetal flow of the actin network in the lab coordinate system is low, and the backward kinematic drift is faster than this flow everywhere in the cell. Under such conditions, the flow in the cell coordinate system is directed to the rear everywhere, and myosin, moving with the actin network, is effectively swept to the rear (see predicted myosin distributions in Figure 5). The predicted adhesion distribution is also high at the rear and low at the front of the cell because the flow sweeps the adhesions to the rear, similar to myosin (see predicted adhesion distributions in Figure 9). Thus, the F-actin density at the rear is damped, while along the front and sides it is distributed in a peaked way (see predicted actin distributions in Figure 9).

At low adhesion strengths and/or when myosin activity is high, the magnitude of the centripetal flow of the actin network in the lab coordinate system is greater, and at the rear it can be faster than the cell speed (especially because the latter slows down under these conditions). The net flow in the cell frame of

reference is directed inward everywhere around the boundary, so myosin, moving with the actin network, is driven inward everywhere in the cell (of course, much more so at the front, only weakly at the rear). Thus, myosin is peaked around the cell body (see predicted myosin distribution in Figure 5). Because of this more central myosin location, it pulls actin inward more evenly though the centripetal flow is still graded faster at the rear, slower at the front, it is graded now smoothly around the boundary (Figure 5). Under these conditions, emerging adhesions as well as F-actin move inward everywhere and pile up close to the cell body (Figure 9A), so around the boundary of the cell, the adhesion distribution remains low, below a threshold at which the F-actin density is damped significantly. The F-actin density stays almost constant around much of the boundary (Figure 9B).

In addition, we simulated actin network flow maps and myosin, adhesion, and actin distributions for the the cases where ζ varies with adhesion density. In the case where ζ decreases with adhesion density (Supplemental Figure 8), the simulated patterns are similar to those simulated under the assumption that ζ is spatially constant and match the experimentally observed patterns. However, in the case where ζ increases with adhesion density, the simulated patterns of retrograde actin flow did not match the experimentally observed patterns (Supplemental Figures 9). Specifically, the rate of retrograde flow was nearly constant around the cell perimeter at intermediate values for ζ, in contrast to the step-like increase in flow observed experimentally (compare Supplemental Figure 9C-D, Figure 7C-D).

Stability of the cell shape

The cell shape evolves dynamically according to the graded vector sum of the outward polymerization and inward centripetal flow at the boundary. The flow, on the other hand, depends on the cell shape. The iterative procedure of evolving the coupled flow and shape showed that the stable steady fan-like shape evolves in the low adhesion case. This shape is very similar to that observed, with the aspect ratio $\sim$1.5. When the adhesion is increased to its strength in the control case, the shape elongates becoming the canoe-like and stabilizes with the aspect ratio ~ 2.5, similar to the observed cell (Figure 11).

References

[1] Janson LW, Kolega J, Taylor DL (1991) Modulation of contraction by gelation/solation in a reconstituted motile model. J Cell Biol 114: 1005–1015.

[2] Carlsson AE (2006) Contractile stress generation by actomyosin gels. Phys Rev E Stat Nonlin Soft Matter Phys 74: 051912.

[3] Rubinstein B, Fournier MF, Jacobson K, Verkhovsky AB, Mogilner A (2009) Actin-myosin viscoelastic flow in the keratocyte lamellipod. Biophys J 97: 1853–1863.

[4] Mitchison T, Kirschner M (1988) Cytoskeletal dynamics and nerve growth. Neuron 1: 761–772.

[5] Chan CE, Odde DJ (2008) Traction dynamics of filopodia on compliant substrates. Science 322: 1687–1691.

[6] Ballestrem C, Hinz B, Imhof BA, Wehrle-Haller B (2001) Marching at the front and dragging behind: differential alphavbeta3-integrin turnover regulates focal adhesion behavior. J Cell Biol 155: 1319–32.

[7] Fournier MF, Sauser R, Ambrosi D, Meister JJ, Verkhovsky AB (2010) Force transmission in migrating cells. J Cell Biol 188: 287–297.

[8] Lee J, Jacobson K (1997) The composition and dynamics of cell-substratum adhesions in locomoting fish keratocytes. J Cell Sci 110 (Pt 22): 2833–2844.

[9] Anderson KI, Cross R (2000) Contact dynamics during keratocyte motility. Curr Biol 10: 253–260.

[10] Hu K, Ji L, Applegate KT, Danuser G, Waterman-Storer CM (2007) Differential transmission of actin motion within focal adhesions. Science 315: 111–115.

[11] Wilson CA, Tsuchida MA, Allen GM, Barnhart EL, Applegate KT, et al. (2010) Myosin ii contributes to cell-scale actin network treadmilling through network disassembly. Nature 465: 373–377.

[12] Lacayo CI, Pincus Z, VanDuijn MM, Wilson CA, Fletcher DA, et al. (2007) Emergence of large-scale cell morphology and movement from local actin filament growth dynamics. PLoS Biol 5: e233.

[13] Keren K, Pincus Z, Allen GM, Barnhart EL, Marriott G, et al. (2008) Mechanism of shape determination in motile cells. Nature 453: 475–480.

[14] Lee J, Ishihara A, Theriot JA, Jacobson K (1993) Principles of locomotion for simple-shaped cells. Nature 362: 167–171.

[15] Schakenraad JM, Busscher HJ, Wildevuur CR, Arends J (1988) Thermodynamic aspects of cell spreading on solid substrata. Cell Biophys 13: 75–91.

[16] Raucher D, Sheetz MP (1999) Characteristics of a membrane reservoir buffering membrane tension. Biophys J 77: 1992–2002.
