# Supplementary material for: An Adhesion-Dependent Switch between Mechanisms That Determine Motile Cell Shape
Source: PLoS Biol. 2011 May 3;9(5):e1001059. doi: 10.1371/journal.pbio.1001059 (PMC3086868; doi:10.1371/journal.pbio.1001059)
Supplement: Text S2 — Supplemental results. (PDF) [file pbio.1001059.s022.pdf]

Text S2

Supplemental Results

The effects of calyculin A on cell speed and area are reduced when myosin contraction is inhibited with blebbistatin

Calyculin A is a phosphatase inhibitor that is thought to indirectly increase myosin II activity [1], and we have found that keratocytes treated with calyculin A migrate at higher speeds and are smaller than control cells (see Figure 8 in the main text). To determine whether these effects are primarily due to increased myosin II activity, we treated cells crawling on glass surfaces with either blebbistatin, a myosin-II inhibitor [2], or calyculin A alone, or both blebbistatin and calyculin A (Supplemental Figure 6). We found that cells treated with blebbistatin were significantly larger and slower than control cells (average area = 424 μm^2 and average cell speed = 0.20 μm/s for control cells; average area = 503 μm^2, p = 0.005, Student's T test, and average cell speed = 0.17 μm/s, p = 0.002, for cells treated with blebbistatin). Cells treated with calyculin A were significantly smaller and faster than control cells (average area = 332 μm^2, p = 0.001 and average cell speed = 0.28 μm/s, p = 10^{-5}). The effects of calyculin A on cell area and speed were greatly reduced when the cells were treated with blebbistatin for 30 minutes prior to calyculin A treatment: cells treated with both blebbistatin and calyculin A were not significantly different from control cells (average area = 405 μm^2, p = 0.44, and average cell speed = 0.19 μm/s, p = 0.38). Although we cannot rule out the possibility that calyculin A affects the activity of signaling proteins that regulate adhesion and motility, these results suggest that the effects of calyculin A on cell shape and speed are primarily due to increased myosin II activity.

References

[1] Ishihara H, Ozaki H, Sato K, Hori M, Karaki H, et al. (1989) Calcium-independent activation of contractile apparatus in smooth muscle by calyculin-a. J Pharmacol Exp Ther 250: 388–396.

[2] Straight AF, Cheung A, Limouze J, Chen I, Westwood NJ, et al. (2003) Dissecting temporal and spatial control of cytokinesis with a myosin ii inhibitor. Science 299: 1743–1747.
